# Phenotypic characterization and complete genome of a tumorigenic pathobiont *Escherichia coli* LI60C3

**DOI:** 10.1186/s13099-025-00732-1

**Published:** 2025-08-20

**Authors:** Linda Chia-Hui Yu, Shu-Chen Wei, Yi-Hsuan Li, Chung-Yen Huang, Yu-Chen Pai, Yuan-Mao Hung, Liang-Chuan Lai, Yen-Hsuan Ni

**Affiliations:** 1 Graduate Institute of Physiology, National Taiwan University College of Medicine, Taipei, Taiwan R.O.C.; 2 Department of Internal Medicine, National Taiwan University Hospital, Taipei, Taiwan R.O.C.; 3 Department of Pathology, National Taiwan University Hospital, Taipei, Taiwan R.O.C.; 4 Bioinformatics and Biostatistics Core, Center of Genomic and Precision Medicine, National Taiwan University, Taipei, Taiwan R.O.C.; 5 Graduate Institute of Biomedical Electronics and Bioinformatics, National Taiwan University, Taipei, Taiwan R.O.C.; 6 Department of Pediatrics, National Taiwan University Hospital, Taipei, Taiwan R.O.C.

**Keywords:** Colorectal cancer microbiome, Tumorigenic *E. coli*, Invasive pathobionts, Comparative genomics, Complete genome sequencing, Intestinal epithelial cells, Phylogenetic tree, Experimental pathobiont models

## Abstract

**Background:**

Symbiotic microbes benefit the host, but the emergence of pathobionts leads to disease. An invasive *Escherichia coli* LI60C3, isolated from mouse colonocytes, shows colitogenic and tumorigenic properties. Despite extensive research on the role of microbiota in colorectal cancer (CRC) development, the genetic markers associated with this pathobiont remain elusive. The objective is to characterize the tumorigenic *E. coli* through whole-genome sequencing (WGS) and phenotypic assays, and validate their presence in human CRC.

**Methods:**

The intracellular bacterial counts and proliferation rates of human intestinal epithelial cells were evaluated after exposure to various *E. coli* strains. Tumor burden was assessed in mice orally administered LI60C3. WGS of LI60C3 was performed on a PacBio Sequel II platform, and the long reads were assembled *de novo* for gene annotation and detection of virulence factors and antibiotic resistance. Bacteria-specific genes were assessed in CRC specimens by qPCR analysis.

**Results:**

A 100-fold increase in intracellular bacterial count was observed in epithelial cells exposed to LI60C3 compared to commensal *E. coli* strains. LI60C3 resulted in a threefold increase in epithelial cell cycle rate and a fourfold rise in mouse tumor numbers. WGS revealed a circular chromosome of 4,863,930 bases for LI60C3, demonstrating a high sequence homology to adherent-invasive *E. coli* LF82 (91%) and NC101 (87%) and to uropathogenic *E. coli* 536 (88%). Two extrachromosomal plasmids, pTra and pCoMb, were identified. While pTra exhibits sequence homology with other commensal *E. coli* plasmids, pCoMb has partial matches with those found in pathogenic bacteria. LI60C3 is classified as phylogroup B2 and expresses virulence factors, including Type 1 and P fimbriae, contact-dependent growth inhibition system, iron acquisition system, and hemolysin. Unique gene clusters, named Epm and Phz islands, were identified in the LI60C3 genome. The emergence of LI60C3-specific genes was observed in mouse tumors induced by chemicals and gene mutation, and higher levels of LI60C3 markers were validated in human CRC specimens compared with healthy mucosal samples.

**Conclusion:**

Genetic signatures of LI60C3 were detected in mouse and human CRC. The comparative genome analysis for LI60C3 helps identify pathobionts and may be used as cancer predictors.

**Supplementary Information:**

The online version contains supplementary material available at 10.1186/s13099-025-00732-1.

## Introduction

Microbiota dysbiosis is associated with developing inflammatory bowel diseases (IBDs) and colorectal cancers (CRC) [1–3]. While symbiotic commensals inhabit the gut lumen in healthy humans, elevated levels of mucosal bacteria were detected in clinical specimens from patients [4–6]. The presence of intraepithelial bacteria in gut tissues was identified in mouse models of colitis and cancers, suggesting microbial etiology of the diseases [7–9]. Meta-analysis and machine-learning algorithms revealed specific microbial markers and distinct microbiome and metabolome profiles associated with colon polyposis and CRC development [10–14]. Growing evidence indicates that pathobionts (commensal-derived opportunistic pathogens) are involved in the onset or perpetuation of diseases, and mucosal bacteria may play a more significant role than fecal bacteria in the pathogenesis associated with microbiota.

*Escherichia coli* is a facultative anaerobe that colonizes the intestine of humans and animals, and constitutes a minor population of the fecal microbiota in a healthy state of gut anaerobiosis. Nevertheless, a high abundance of *E. coli* was found in the gut tissues of patients with IBDs, colorectal adenoma and carcinoma, and familial adenomatous polyposis (FAP) [15–20], suggesting that mucosa-associated bacteria were involved in inflammation and tumorigenesis. We previously demonstrated that an invasive *E. coli* strain isolated from intestinal epithelial cells, designated LI60C3, increased colitis susceptibility and promoted cancer initiation in mouse models [21, 22]. Direct actions of LI60C3 on epithelial cell functions were shown in primary colonoid cultures and human Caco-2 cell lines [21, 22]. These studies revealed that LI60C3 accelerated cell cycle progression, enhanced spheroid growth, increased cytokine synthesis, and disturbed the circadian rhythm of epithelial cells in vitro [21, 22]. These findings underscore the tumorigenic potential of LI60C3, highlighting its capacity to influence epithelial cell behavior independently of immune cell activation. These unique features of LI60C3 prompted us to conduct further genomic characterization of this tumorigenic pathobiont. Other laboratories found adherent-invasive *E. coli* (AIEC) and diffusely adherent *E. coli* (DAEC) linked to inflammatory and diarrheal diseases, with colitogenic effects shown in experimental models through orogavage administration [23–25]. Additionally, colibactin-producing *E. coli* have been identified for their roles in causing DNA damage and cell senescence in epithelial cells in vitro, as well as promoting severe colitis and invasive tumor growth in chemically induced and genetically modified mice [26–33]. Although AIECs and DAECs were initially isolated from patients, they also exist in a quarter of healthy individuals [34–38]. Currently, the virulence characteristics of intestinal pathobionts are mainly assessed through phenotypic assays. The genetic differences between commensals and pathobionts are still not well understood.

In contrast to commensal and pathobiont *E. coli* that persistently colonize the gut, pathogenic *E. coli* strains are accountable for acute infections in the enteric, urinary, and respiratory tracts, and the nervous system. The *E*. *coli* pathotypes causing foodborne diarrheal diseases consist of enteropathogenic *E. coli* (EPEC), enterohaemorrhagic *E. coli* (EHEC) (a subset of Shiga toxin-producing *E. coli*), heat-stable and -labile toxin-producing enterotoxigenic *E. coli* (ETEC), enteroinvasive *E. coli* (EIEC), and enteroaggregative *E. coli* (EAEC) [39]. Other *E. coli* pathotypes can cause extra-intestinal diseases, including uropathogenic *E. coli* (UPEC), which is responsible for cystitis, pyelonephritis, septicemia, and meningitis [40]. Pathogenic strains of *E. coli* possess a variety of virulence factors, which include adherence and invasion determinants, enterotoxins, and multiple secretion systems. These systems facilitate the injection of bacterial components into eukaryotic cells, leading to the manifestation of symptoms in the host.

Despite the pathogenic traits of LI60C3, the genetic markers and virulence factors to distinguish the bacteria from commensal *E. coli* strains remain unresolved. Virulence factors are encoded by a cluster of genes in a pathogenicity island localized in the chromosome or extrachromosomal plasmids. Bacteria use plasmids for horizontal gene transfer to enhance virulence and acquire toxin production and invasive capabilities. Increased expression of fimbrial adhesion (fimbriae/pili) and genes related to stress survival responses (high temperature requirement (htr) A) were detected in LI60C3 following repeated internalization in epithelial cells, which correlated with their higher levels in mouse fecal samples during the tumor transition phase from dysplasia to carcinoma [21]. Nevertheless, fimbriae and htrA proteases are not specific to the LI60C3 strain but exist in many *Enterobacteriaceae* genera. In this regard, virulence factors and genetic signatures of the tumorigenic LI60C3 await revelation. Investigating specific markers of the tumorigenic LI60C3 through the integration of genomic, phenotypic, and in vivo approaches may aid in clinical treatment and facilitate non-invasive CRC screening.

This study examines the phenotypic characteristics of LI60C3 using bacteria-epithelial cocultures and mouse tumor models. Whole genome sequencing (WGS) was performed to decipher the genetic signatures of LI60C3. Here, we report the *de novo* assembly of a circular chromosome and two extrachromosomal plasmids for the LI60C3 strain. The pathobiont-specific biomarkers identified through genome comparison between LI60C3 and reference *E. coli* strains were evaluated in mouse tumor models and human CRC specimens.

## Materials and methods

### Animal study

Experimental procedures in mouse models were approved by the IACUC Committee of NTUCM (IACUC#20210023, IACUC#20230363). Specific pathogen-free BALB/c mice and Apc(Min/+) mice were purchased from the National Institutes of Applied Research and kept in temperature-controlled rooms (23 ± 2 °C) with 12 h light-dark cycles. Mice were fed standard chow and water *ad libitum*. At the end of the experiments, mice were injected with pentobarbital (200 mg/kg) overdose for laparotomy and inspection of intestinal tumors. Regular monitoring of animals for signs of distress and significant weight loss with welfare-based criteria (e.g., lethargy, inability to reach food and water), and timely intervention are applied as humane endpoints.

### Bacterial strains

The bacterial colonies were stored in 50% glycerol at -80 °C until use, and only the first three generations after Luria-Bertani (LB) broth culturing were used as stocks. Glycerol stocks of *E. coli* strains were inoculated into LB broth for culturing at 37 °C with constant shaking overnight. The bacterial culture was plated on LB agar to grow overnight, and a single colony was then inoculated into LB broth for culturing at 37 °C to mid-exponential phase (OD_600_ at 0.6–0.8). The *E. coli* LI60C3 strain was isolated from colonic epithelial cells (colonocytes) of a mouse colitis-associated cancer model [21, 22]. Other bacteria included the laboratory K-12 strains of *E. coli* (i.e., MG1655, DH5α, and JM109) or strains isolated from mouse colonocytes in colitis and cancer models (e.g., HM784C1, HM926C2, and HM936C4) [21, 22]. The Crohn’s-associated AIEC strain LF82 [16, 23, 41] was from Dr. Shu-Chen Wei, Department of Internal Medicine of NTUCM. The mouse AIEC strain NC101 [27, 42, 43] was a kind gift from Dr. Christian Jobin, Department of Medicine, University of Florida College of Medicine.

### Chemically induced mouse tumor model

Mouse models of colitis-associated CRC were prepared according to previously established methods [44, 45]. Briefly, BALB/c mice (6–8 weeks old) were randomly divided into two experimental groups (*N* = 8–10/group). One group was injected intraperitoneally with azoxymethane (AOM) (10 mg/kg body weight) and after 7 days, given 2% dextran sodium sulfate (DSS) in drinking water for 4 days, followed by 3 days of regular water (AOM/DSS). The other group was age-matched mice that were not administered agents and used as untreated controls. Three cycles of AOM/DSS were administered for tumor induction. Our previous studies have shown high numbers of colon tumors in the AOM/DSS-induced mice 56–90 days after the first AOM injection [21, 44, 45].

In another setting, BALB/c mice (6–8 weeks old) were randomly divided into three experimental groups (*N* = 8–10/group). The mice subjected to AOM/DSS cycles were administered ABX mixtures (vancomycin 500 mg/L, neomycin 1 g/L, metronidazole 1 g/L, and ampicillin 1 g/L) in drinking water from day 56–63 to disturb gut microbiota, followed by orogastric administration of either phosphate-buffered saline (PBS), LI60C3, or LF82 strain at 10^9^ CFU per mouse on day 64. At the end of the experiments, the entire intestine was examined macroscopically under a dissecting microscope to quantify the number and size of tumors on day 90. Tumor area was assessed using imaging software (AxioVision Imaging System, Zeiss). Paraffin-embedded tissues were used for histological assessment and tumor grading by a pathologist blinded to the treatment groups (see below).

### Genetic mutant Apc(Min/+) mouse model

The Apc(Min/+) mice is a well-established animal model bearing multiple intestinal neoplasia (Min), that simulates human FAP and CRC [42, 46]. The adenomatous polyposis coli (APC) gene is a key tumor suppressor gene frequently mutated in the early stages of human CRC. Mice with a single allele of the *Apc* gene mutation were generated by in vitro fertilization (IVF) of C57BL/6 oocytes with Apc(Min/+) sperm in the National Institutes of Applied Research in Academia Sinica, Taipei. Our pilot studies by intercrossing of Apc(Min/+) males with C57BL/6 females showed high mortality of the offspring, and the breeding strategy of heterozygotic pairs was also unfeasible due to infertility of the female Apc(Min/+) mice. The IVF embryos were transferred to surrogate CByB6F1 dams (a cross-strain of C57BL/6 and BALB/c mice), and the offspring of Apc(Min/+) and wild-type (WT(+/+)) littermates were born in a 1:1 ratio. The WT(+/+) and Apc(Min/+) mice were confirmed by genotyping (*N* = 10/group), and tumor development was observed by macroscopic inspection at 20 weeks of age. Paraffin-embedded tissues were used for histological assessment and tumor grading by a pathologist blinded to the treatment groups (see below).

### Tumor assessment criteria

The intestinal tissues were fixed in 4% paraformaldehyde and processed for paraffin embedding. The sections stained with hematoxylin and eosin dyes (H&E) were used for histopathological examination and tumor grading by a blinded pathologist [44, 45]. Tumor morphology was classified as low-grade dysplasia, high-grade dysplasia, and carcinoma. Low-grade dysplasia is characterized by disorganized crypt structures and monolayered, hyperchromatic epithelial cells. High-grade dysplasia is characterized by disoriented crypts lined with stratified epithelial cells, where the glands appear back-to-back with limited seperation, and the cells exhibiting high nuclear to cytoplasmic ratio and loss of cellular polarity. Carcinoma is defined as the loss of crypt architecture and the penetration of the dysplastic cells through the basement membrane.

#### Human cell lines

Human colorectal adenocarcinoma Caco-2 cell lines, which exhibit monolayer features, were employed for the bacteria-epithelial cocultures. The cells were purchased from the ATCC/Bioresource Collection and Research Center (BCRC), and an STR-PCR profile was performed by BCRC. The Caco-2 cells were grown in Dulbecco’s Modified Eagle’s Medium (DMEM) (Cat. #11885-076, Gibco brand, ThermoFisher Scientific, Waltham, MA, USA). The medium was supplemented with 10% fetal bovine serum (FBS) (Cat. #26140-079, Gibco), 100 U/ml penicillin and 0.1 mg/ml streptomycin (Cat. #P4333, Sigma-Aldrich, St. Louis, MO, USA), and 15 mM HEPES (Cat. #26140-079, Gibco). The Caco-2 (C2BBe clone) was also supplemented with human holo-transferrin (11 mg/l) (Cat. #T0665, Sigma). The cells were grown to confluency at 37 °C with 5% CO_2_ in a humidified incubator for experiments [47, 48].

#### Bacterial internalization in epithelial cell cultures

The quantity of bacteria within epithelial cells was measured as described [49, 50]. Briefly, Caco-2 cells were seeded in 12-well plates at a concentration of 3 × 10^4^ cells/ml and cultured for 7 days to reach confluency (4 × 10^5^ cells per well). After removal of the antibiotic-containing culture media, the cells were rinsed with sterile PBS twice and incubated in antibiotic-free culture media for 1 h. The Caco-2 cells were then exposed to bacteria in antibiotic-free culture media at multiplicity-of-infection (MOI) = 10 and incubated for 4 h at 37 °C. For the groups without bacteria, PBS was added as a vehicle control. The bacterial numbers in the apical solution and inside the cells were determined by plating apical culture media or cell lysates on LB agar plates for CFU counting. To obtain intracellular bacterial counts, the monolayer was rinsed with sterile PBS twice and incubated with PBS containing 0.25% Trypsin/EDTA (Cat. #25200, ThermoFisher Scientific) for 10 min at 37 °C. The epithelial cells were centrifuged at 300 × *g* for 3 min, washed with PBS, and centrifuged again. The cells were then resuspended in a PBS solution containing 300 µg/ml gentamicin (Cat. #VGE1AC04, Taiwan Biotech Co., Taoyuan, Taiwan) for incubation at 37 °C for 1 h. After cell lysis with 200 µl of 1% Triton X-100 (Cat. #X100, Sigma-Aldrich) in PBS for 10 min on ice, the lysate was plated on LB agar plates at 37 °C overnight. The number of viable intracellular bacteria was expressed as log_10_CFU/10^6^ cells.

#### Flow cytometric analysis of cell cycle rates

Human cell lines exposed to bacteria for 4 h were rinsed with PBS twice and incubated in culture medium containing 300 µg/ml of gentamicin for 1 h. After this initial incubation, the medium was replaced with fresh culture medium containing 300 µg/ml of gentamicin, and the cells were allowed to incubate for up to 24 h to evaluate cell proliferation, following established protocols [44, 45]. The monolayered cells was rinsed with sterile PBS twice, and incubated with PBS containing 0.25% Trypsin/EDTA for 10 min at 37 °C to obtain single cells. After centrifugation at 300 × *g* for 3 min and supernatant removed, the cells were washed with PBS and centrifuged again. The cells were then fixed with ice-cold 70% ethanol overnight, followed by washing with PBS containing 1% bovine serum albumin (BSA) (Cat. #A2153, Sigma-Aldrich) and centrifuged at 1500 × *g* for 5 min. After decanting the supernatant, the cells were stained with a primary antibody rabbit anti-human Ki67 (Cat. #9129) (1:400, Cell Signaling, Beverly, MA, USA) in PBS containing 0.5% TWEEN-20 (Cat. #TWN508, BioShop, Burlington, ON, Canada) and 1% BSA for 1 h at room temperature. The cells were rinsed with 1% BSA in PBS, and then incubated with a secondary antibody, goat anti-rabbit IgG conjugated to Alexa-Fluor 488 (Cat. #A11034) (1:500, Life Technologies Inc., Gaithersburg, MD, USA), in PBS containing 0.5% TWEEN-20 and 1% BSA for 30 min at room temperature. After washing with 1% BSA in PBS, the cells were stained with a propidium iodide (PI) solution in PBS containing 20 µg/ml PI (Cat. #P4170, Sigma-Aldrich) and 200 µg/ml RNase A (Cat. #RNA888, BioShop) for 30 min at room temperature in a light-protected container. The cells were washed again with 1% BSA in PBS, and collected after centrifugation. A minimum of 10,000 PI-stained nuclei were analyzed by flow cytometry (FACS Calibur, BD Life Sciences, Franklin Lakes, NJ, USA), and the percentages of cells in the G0–G1, S, and G2–M phases of the cell cycle and the ratio of cells with high to low intensity of Ki67 were determined using Mod Fit LT cell cycle analysis software (Verity Software, Topsham, ME, USA) and FlowJo software (BD Life Sciences).

#### Whole genome sequencing, data post-processing, and de Novo assembly

The complete genome of *E. coli* strain LI60C3 was obtained by third-generation sequencing using a Pacific Biosciences (PacBio) Sequel II platform (Taiwan Genomic Industry Alliances Inc., Taipei, Taiwan). Bacterial DNA was extracted using a Qiagen Genomic-tip 20/G kit, and the purity of nucleic acid preparation was determined by Qubit^®^ fluorometer (ThermoFisher Scientific) with High Sensitivity dsDNA assay kits. Each sample was quantified in quadruplicate. Pure DNA was designated with an A260/280 absorbance ratio of 1.8, and an A260/230 ratio of 2.0 or higher to pass the quality control.

Continuous long-read (CLR) datasets generated by the PacBio platform were used for *de novo* assembly of the whole genome with SMRT Link Software (v. 10.1.0.119588). The parameters of predicted accuracy (Phred Scale) are set to 20, genome length to 5 M, maximum plasmid size to 300,000, and downsampled coverage to 100. The depth of coverage plots and the GC coverage plots were produced using the built-in software (corrected error rate = 0.045, cor out coverage = 40). The obtained draft assembly was polished by removing redundant sequences and correcting raw reads using overlaps with other reads. The core polishing uses the arrow algorithm, a left-right Hidden Markov-Model (HMM) that models the enzymatic and photophysical events in SMRT Sequencing. Polished contigs with the origin of replication (ori) for rotation and header adjustment were applied before the sequence submission to a public database. The contigs generated by the long-read sequences, including a circular chromosome and two plasmids, in the LI60C3 genome were deposited in GenBank under the accession numbers CP136900, CP136901, and CP136902.

#### Genome annotation and comparative genomic analysis

The bacterial sequences were annotated using a gene prediction tool Prokka (v1.14.6). The genome sequences were compared to other *E. coli* strains published in the context of the ColiScope project [51] by using the basic local alignment search tool (BLAST) in NCBI (www.ncbi.nlm.nih.gov/BLAST/). The mapping rates were calculated by using the quality assessment tool for genome assemblies (QUAST) (https://quast.sourceforge.net/quast) [52]. Moreover, the conserved gene clusters, i.e., synteny groups, were computed and plotted using XMatchView (https://github.com/bcgsc/xmatchview). The circular map of the bacterial genome was drawn using pyCircos (https://github.com/ponnhide/pyCircos).

#### Virulence and resistance genes prediction

Virulence factor-encoding genes were predicted based on the curated virulence factor database (VFDB) v. 2022 (http://www.mgc.ac.cn/VFs/) [53, 54]. Based on the complete genome FASTA sequence, the coding region was predicted using a gene prediction tool GLIMMER3 applied in the VFanalyzer. The official tool provided by the VFDB developers, VFanalyzer, predicts virulence genes through whole-genome ortholog grouping, hierarchical and iterative homologous searches, and context-based data refinement. Analyses were performed using default settings, including internally defined thresholds for gene identity and coverage.

Resistance genes were predicted by the Resistance Gene Identifier (RGI) (v.6.0.3) which performed homolog detection using DIAMOND in the Comprehensive Antibiotic Resistance Database (CARD) (v.3.3.0) (https://arpcard.mcmaster.ca) [55]. We set the select criteria in RGI to be perfect and strict hits only, corresponding to a sequence identity of ≥ 95% and coverage of ≥ 80%.

#### Phylogenetic analysis of E. coli strains

The phylogenetic analysis was based on the sequences of seven housekeeping genes (*arcA*,* aroE*,* icd*,* mdh*,* mtlD*,* pgi*, and *rpoS*) of *E. coli* reference strains to compare with the new isolates. The sequences of the seven housekeeping genes of each *E. coli* strain were subjected to MUltiple Sequence Comparison by Log- Expectation (MUSCLE) analysis. The multiple sequence alignment (MSA) methods refer to a series of algorithmic solutions for the alignment of evolutionarily related sequences, while taking into account gene mutations, insertions, deletions, and rearrangements under certain conditions. Gblocks (https://bio.tools/gblocks) for trimming the MSA was performed, and the genetic tree was drawn using Molecular Evolutionary Genetics Analysis version 11 (MEGA11) software (https://www.megasoftware.net/) [56]. A neighbor-joining method for metric clustering was used to determine the length of the branches between two taxa. The evolutionary distances were computed in the units of the number of amino acid substitutions per site, and are defined as the fraction of mismatches at aligned positions, with gaps either ignored or counted as mismatches.

#### Sanger sequencing

The bacterial DNA was extracted from a single colony on LB agar plates. The plasmid DNA was purified by using a Presto^®^ Mini Plasmid kit, whereas the bacterial chromosome DNA, which may also contain plasmids, was extracted by using a VIOGENE Geno Plus Mini kit. PCR amplification was performed by the First Core Laboratory of NTUCM for Sanger sequencing. PCR conditions were as follows: 95◦C for 3 min, followed by 35 cycles of 95 °C for 30 s, 58 °C for 30 s, and 72 °C for 2 min, and a final elongation step at 72 °C for 5 min. The annealing temperatures were different depending on the target genes.

#### Semi-quantitative, quantitative, and triplex polymerase chain reaction (PCR) methods

Genomic DNA extracted from *E. coli* was subjected to PCR amplification. The *E. coli* phylotyping was performed using Clermont’s triplex PCR analysis by semi-quantitative methods [57]. The semi-quantitative PCR primers for bacterial genes are listed (Suppl Table 1). The PCR products containing Nucleic Acid Stains (Thermo Scientific) were separated on a 1% agarose gel, and visualized by ultraviolet transillumination.

Quantitative real-time PCR (qPCR) analysis was performed using an Applied Biosystems StepOnePlus Real-Time PCR Systems (Applied Biosystems, MA, USA). The qPCR primer pairs for analysis of bacterial genes are listed (Suppl Table 2), including the universal bacterial marker *16S rRNA* gene and the *E. coli-*specific *uidA* gene [58]. Moreover, the forward and reverse primers of the eukaryotic *18S rRNA* gene (*F*: 5’-TCAACTTTCGATGGTAGTCGCCGT-3’, and *R*: 5’-TCCTTGGATGTGGTAGCCGTTTCT-3’) were also used to generate a 108-bp fragment for normalization in some cases. The PCR reaction mixture consisted of 50 ng of RT product, 10 µL of Power SYBR Green PCR Master Mix and 125 nM specific primer pairs in a final reaction volume of 20 µL. The PCR conditions were 95 °C for 10 min, followed by 40 cycles of denaturation at 95 °C for 15 s, and annealing and extension at 60 °C for 1 min. Amplification plots were obtained by using a StepOnePlus™ Software v2.3 (Applied Biosystems). Each sample was run in duplicate, and the mean threshold cycle (CT) was determined from the two runs. The data analysis was based on the comparative threshold method. The CT value of the target genes was subtracted from that of *16 S rRNA* gene as the housekeeping gene (ΔCT). The difference of the ΔCT values between the treatment and the control conditions was determined as the double delta CT value (ΔΔCT). The relative gene expression was calculated as the logarithm of the value of -ΔΔCT to base 2 (2^− ΔΔCT^).

#### Human sample collection for bacterial gene analysis

Colonic specimens were collected from 49 healthy individuals (21 males and 28 females, age interval: 33–78 years) and 114 patients with CRC (65 males and 49 females, age interval: 26–88 years) for analysis in NTUH. The numbers of patients at tumor stages I, II, III, and IV were 20, 39, 33, and 22, respectively. Written informed consent was obtained from all study subjects, and approval for the research ethics of this study was granted by the Institutional Review Board (IRB) for NTUH (#202104001RINC). The tissue RNA was reverse-transcribed and analyzed by qPCR primers of bacterial genes.

### Statistical analysis

Data are presented as mean ± SEM (standard error of mean), unless otherwise stated. The normality of the data was assessed using the Shapiro–Wilk test. When the normality assumption was appropriate, data comparisons were made by unpaired Student’s *t*-test for two groups and one-way analysis of variance (ANOVA) followed by Tukey’s multiple comparison post-hoc tests for three or more groups (GraphPad Prism v. 8.0.1). For data that failed the normality test, non-parametric analysis was employed. Mann–Whitney U test was used for two-group comparisons, and the Kruskal–Wallis test was used for comparisons among three or more groups, followed by Dunn’s multiple comparisons test as a post hoc analysis. A *P* value less than 0.05 is considered significant.

## Results

### Phenotypic characteristics of *E. coli* LI60C3 with invasive and tumorigenic ability

**Fig. 1:**
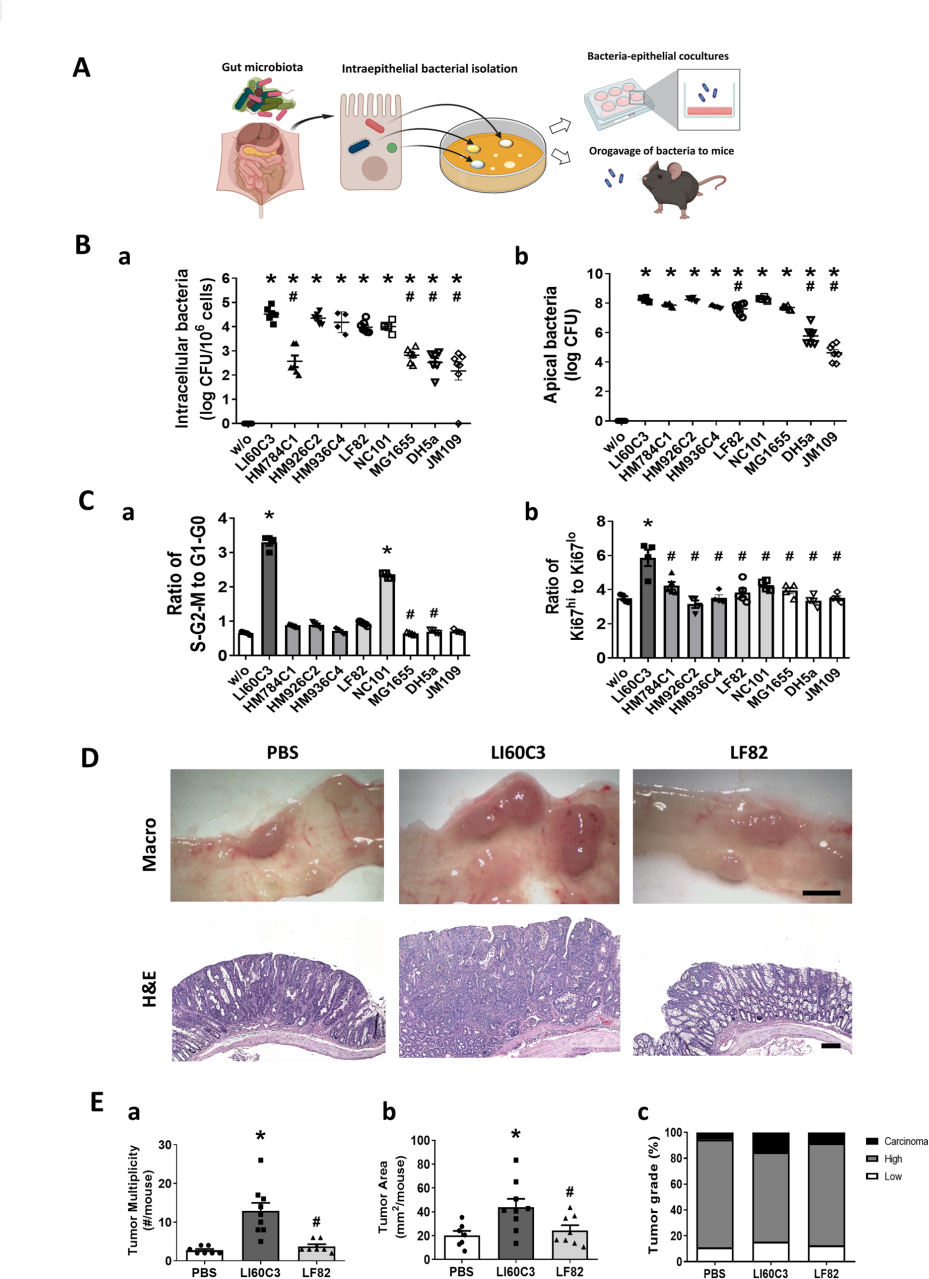
Phenotypic characterization of the invasive feature and tumorigenic ability of *E. coli* strains. **(A)** Schematic diagram of microbial isolation from intestinal epithelial cells for in vitro and in vivo phenotypic assays. The invasive features, proliferation-inducing ability, and tumorigenic characteristics of the intraepithelial bacteria obtained from mouse colonocytes were evaluated in human cell lines and experimental murine models. **(B)** The intraepithelial *E. coli* strains (LI60C3, HM784C1, HM926C2, and HM936C4), adherent-invasive *E. coli* strains (LF82 and NC101), and nonpathogenic laboratory strains (MG1655, DH5α and JM109) were exposed to Caco-2 cells at MOI = 10 for 4 h. (a) Intracellular bacterial counts and (b) apical bacterial counts in the epithelial cultures are shown. **(C)** Cell cycle progression of Caco-2 cells after bacterial exposure for 24 h. The cell ratios in (a) S/G2/M to G1/G0 phase and (b) high to low Ki67 staining intensity are shown. Data are expressed as mean ± SEM (*N* = 5–8/group). Each dot represents the data of one sample. *P < 0.05 *vs.* w/o, and ^#^*P* < 0.05 *vs.* LI60C3. **(D)** A murine model with chemically induced colon cancer was inoculated with PBS vehicle, LI60C3, or LF82 at 10^9^ CFU per mouse via the orogastric route. Representative images of intestinal tumors in each mouse group are displayed. Bar: 2 mm (macroscopic images) and 200 μm (histological images, H&E staining). **(E)** Increased tumor load was observed in mice inoculated with LI60C3, but not LF82. The tumor (a) number, (b) area, and (c) grade were determined for each mouse. The percentages of tumors with low- and high-grade dysplasia and carcinoma in each group are presented. Data are expressed as mean ± SEM (*N* = 8–10/group). Each dot represents the data of one mouse. *P < 0.05 *vs.* PBS, and ^#^*P* < 0.05 *vs.* LI60C3. The statistical significance of all panels is calculated by ANOVA followed by Tukey’s multiple comparison test, except those of (B-a), (C-a) and (E-a) are determined by the Kruskal–Wallis test. Experiments were repeated at least twice.

Our previous studies have demonstrated that the *E. coli* strain LI60C3, isolated from mouse colonocytes, exacerbates colitis severity and increases tumor burden in mouse models [21, 22]. A schematic diagram of the isolation of intraepithelial bacteria for phenotypic characterization is illustrated in Fig. 1A. The invasive characteristics of LI60C3 were compared with those of other *E. coli*, including the adherent-invasive and laboratory strains, using in vitro bacteria-epithelial cocultures. The bacteria were exposed to human Caco-2 cell monolayers at an MOI of 10 for 4 h, and the intracellular bacteria were quantified using a gentamicin resistance assay. The intracellular bacterial counts of LI60C3 were 100 times higher than the nonpathogenic laboratory K-12 strains of *E. coli*, such as MG1655, DH5α, and JM109 (Fig. 1B). Moreover, the apical bacterial counts of LI60C3 also exceeded those of the laboratory strains, indicating a greater extracellular multiplicity or viability of LI60C3 (Fig. 1B). The invasive ability of LI60C3 was comparable to that of the prototypic AIEC strains LF82 and NC101 (Fig. 1B). The LF82 strain was isolated from the ileal lesion of a patient with Crohn’s disease and had strong colitogenic ability in transgenic mice overexpressing human carcinoembryonic antigen-related cell adhesion molecule (CEACAM) 6 ^23, 41^. The NC101 strain carries a pathogenicity island encoding genotoxins with colitogenic and tumorigenic ability in mouse models [27, 42, 43]. Although several *E. coli* strains displayed similar levels of invasiveness, exposure to LI60C3 exerted faster epithelial cell cycle rates compared to the other bacteria (Fig. 1C), suggesting that LI60C3 has a stronger ability to induce epithelial proliferation. Orogastric administration of LI60C3 resulted in an increase in the number and area of colon tumors in mouse models when compared to the LF82 and PBS vehicle groups (Fig. 1D-E).

### Genome comparison of LI60C3 to reference *E. coli* strains

**Fig. 2:**
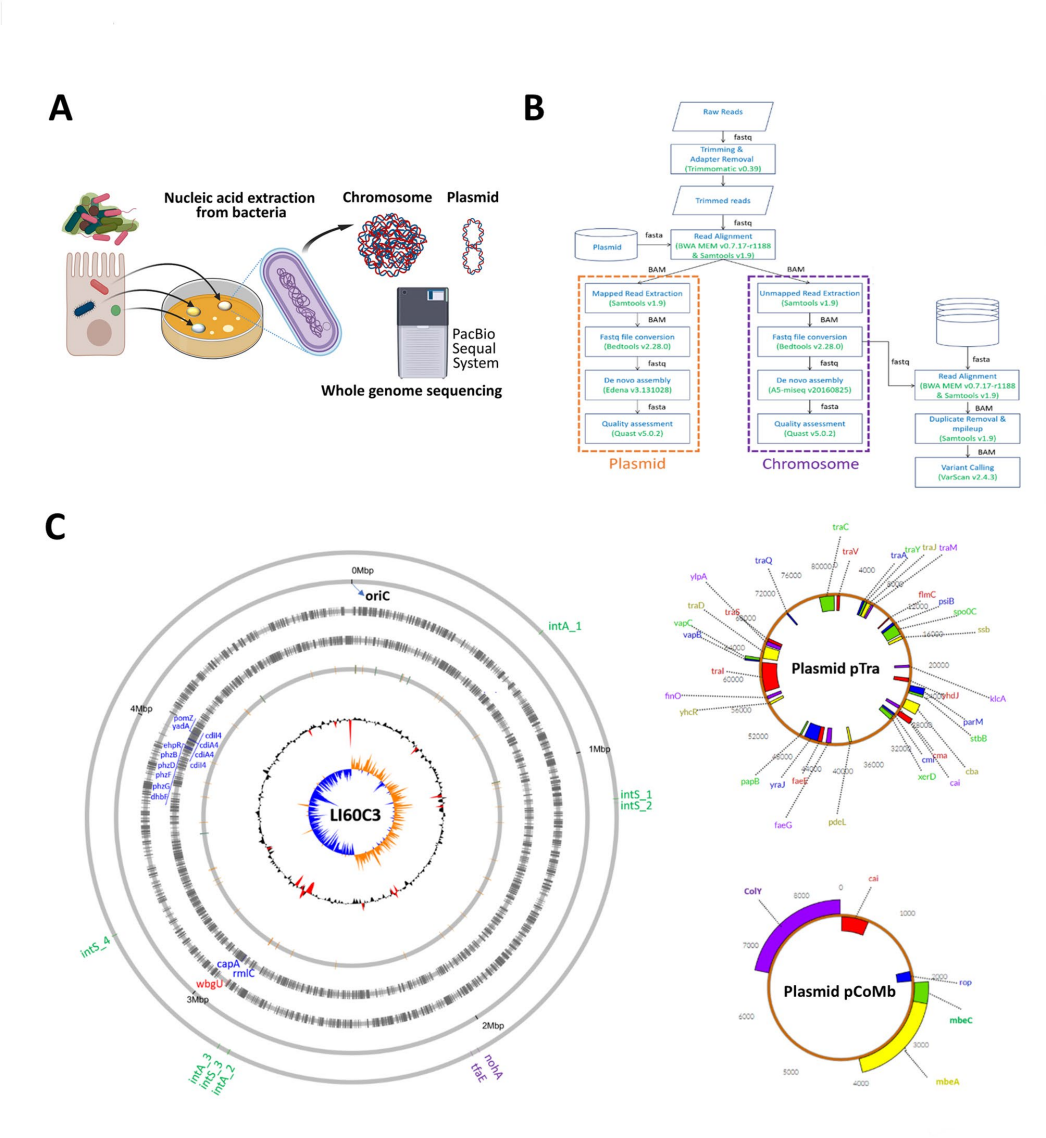
Whole genome sequencing of the tumorigenic *E. coli* strain LI60C3. **(A)** Schema of nucleic acid extraction from LI60C3 for genome sequencing using a PacBio Sequel II platform. The continuous long-read datasets generated by PacBio platform were used for *de novo* assembly of the whole genome. **(B)** Analysis flow chart for quality assessment, trimmed coverage, and contig assembly. **(C)** The bacterial genome was assembled *de novo* through long reads and revealed a circular chromosome (4,863,930 bases) and two extrachromosomal plasmids pTra (81,809 bases) and pCoMb (8,556 bases). Circular maps of *E. coli* LI60C3 chromosome are drawn starting at the origin of replication (*oriC*). From the outside in, the first circle shows the locations of phage integrases (green) and prophage-like genes (purple). Circle 2 shows the nucleotide sequence positions (in Mbp). Circles 3 and 4 show protein coding sequences (CDSs) transcribed clockwise and anticlockwise, respectively (genes conserved among LI60C3 and the other twelve *E. coli* reference strains (grey); unique cluster genes of LI60C3 transcribed clockwise (red); unique cluster genes of LI60C3 transcribed anticlockwise (blue)). Circle 5 shows the tRNA genes (brown) and rRNA genes (light blue). Circle 6 shows a plot of GC content in a 10-kb window. (mean GC content in a 10-kb window– overall mean GC). Red areas indicate that the deviation is higher than 2 Standard deviations. Circle 7 shows a plot of GC skew ([G-C]/[G + C], in a 10-kb window). Orange areas indicate that G > C, and blue areas indicate that G < C. Moreover, two plasmids were found in the LI60C3 genome, including pTra and pCoMb. The boxes in the outer and inner circles represent CDSs transcribed clockwise and anticlockwise, respectively

To understand the virulent strategy of LI60C3, the bacterial genome was analyzed using third-generation sequencing. Table 1 summarizes the key features of the *E. coli* LI60C3 strain and the genome sequencing methods. The experimental design for nucleic acid extraction from intraepithelial bacteria and the analysis workflow are presented (Fig. 2A and B). The bacterial genome was assembled *de novo* through CLR datasets generated by the PacBio sequencing into one contig, revealing a circular chromosome of 4,863,930 bases (Fig. 2C). The bacterial chromosome replication, which occurs at the origin of replication (*oriC*), is marked on the circular gene map. Moreover, two extrachromosomal circular plasmids with the lengths of 81,809 bases and 8,556 bases were identified by long-read assembly and were named pTra and pCoMb, respectively (Fig. 2C). Based on the final polished assembly data, there are 4464 coding sequences (CDSs), 88 transfer RNA (tRNA), 22 ribosomal RNA (rRNA), 1 transfer-messenger RNA (tmRNA), and 1 repeat region in the LI60C3 chromosome.

**Table 1:** Analysis of *Escherichia coli* LI60C3 strain

Items	Descriptors
**Investigation type**	Bacteria
**Project name**	Whole genome sequencing of *Escherichia coli* LI60C3
**Isolation sources**	Mouse colonic epithelial cells
**Isolation date**	2017.06.26
**Estimated length** **in bases**	4,863,930 (chromosome), 81,809 and 8,556 (plasmid)
**Sequencing method**	PacBio Single Molecule Real-Time (SMRT) sequencing
**Sequencing format**	continuous long reads (CLR)
**Assembly**	SMRT link
**Assembly level**	1 contig (chromosome), 2 contigs (2 plasmids)
**Circular**	yes
**Polymerase Read bases**	3,182,269,527
**Polymerase Read counts**	45,050
**Polymerase Read length (mean)**	70,638
**Subread counts**	346,106
**Subread length (mean)**	9,129
**Subread N50**	10,398
**Trimmed coverage**	576x, 1046x, 766x
**No. of coding sequences**	4464, 89, 8
**No. of tRNA**	88, 0, 0
**No. of rRNA**	22, 0, 0
**GC content**	50.7%, 51.3%, 47.5%
**Finishing strategy**	Sequencing & *de novo* Assembly

The complete genome of LI60C3 was compared with those of reference *E. coli* in public databases, which included two commensal strains, four pathobiont strains, and six pathogenic strains (Table 2). The LI60C3 gene sequence was aligned with those of nonpathogenic laboratory strains of *E. coli*, such as MG1655 and W3110. The genome of K-12 substrains (i.e., MG1655 and W3110) derived from human stool with minimal genetic manipulation was used for comparison, whereas those engineered for plasmid transfection efficiency were not (e.g., DH5α and JM109). The chromosome sequence of LI60C3 exhibited mapping rates of 83.36% and 83.31%, in comparison to MG1655 and W3110 (Table 2).

**Table 2:** Genome comparison of *Escherichia coli* LI60C3 to reference strains

Bacteria (Pathotype)	Serotype	Phylo- group	Source, Human or mouse	GenBank Accession no.	Length in bases	Mapping rate (%) vs. LI60C3 or pTra and pCoMb
**K12 MG1655** (nonpathogenic)	OR: H48:K-	A	Feces, a healthy subject	U00096.3	**4**,**641**,**652 (chromosome**,** circular)**	**83.36%**
**K-12 W3110** (nonpathogenic)	Not defined	A	Feces, a healthy subject	AP009048.1	**4**,**646**,**332 (chromosome**,** circular)**	**83.31%**
**LF82** (AIEC)	O83:H1	B2	Ileum, a patient with Crohn’s disease	CU651637	**4**,**773**,**108 (chromosome**,** circular)**	**91.00%**
				CU638872	108,379 (plasmid, pLF82)	0 and 0%
**NC101** (AIEC)	O2:H6/41	B2	Cecum, wild-type 129S6/SvEv mice	CP072787	**5**,**030**,**087 (chromosome**,** circular)**	**86.97%**
**SK1144** (DAEC)	Not defined	B2	Feces, a healthy subject	NZ_AP018784.1	**5**,**029**,**244 (chromosome**,** circular)**	**83.93%**
				NZ_AP018785.1	169,561 (plasmid, pSK1144)	22.63 and 0%
**2017 C-4173W12** (DAEC)	O^−^:H2	D	Feces, a patient with diarrheal disease	NZ_CP030768.1	**5**,**300**,**308 (chromosome**,** circular)**	**75.53%**
				NZ_CP030770.1	149,680 (plasmid, p2017C-4173W12)	0 and 0%
				NZ_CP030769.1	27,128 (plasmid, pMCR-1_2017C-4173W12)	24.32 and 0%
**E2348/69** (EPEC)	O127:H6	B2	Feces, a patient with diarrheal disease	NC_011601	**4**,**965**,**553(chromosome**,** circular)**	**82.95%**
				NC_011603	97,978 (plasmid, pMAR2)	37.18 and 0%
				DQ388534.1	101,558 (plasmid, pMAR7)	35.76 and 0%
**CFSAN029787** (EIEC)	O96: H19	B1	Feces, a patient with diarrheal disease	NZ_CP011416.1	**4**,**947**,**515 (chromosome**,** circular)**	**78.66%**
				NZ_CP011417.1	293,826 (plasmid, pCFSAN029787_01)	11.93 and 0%
				NZ_CP011418.1	47,606 (plasmid, pCFSAN029787_02)	23.39 and 0%
**EDL933** (EHEC/STEC)	O157:H7	E	Feces, a patient with diarrheal disease	NZ_CP008957.1	**5**,**547**,**323(chromosome**,** circular)**	**69.67%**
				NZ_CP008958.1	92,076 (plasmid, pEDL933)	19.59 and 0%
**H10407** (ETEC)	O78:H11	A	Feces, a patient with diarrheal disease	NC_017633.1	**5**,**153**,**435 (chromosome**,** circular)**	**76.32%**
				NC_017724.1	94,797 (plasmid, p948)	41.63 and 0%
				NC_017722.1	66,681 (plasmid, p666)	4.09 and 0%
**536** (UPEC)	O6:K15:H31	B2	Urine, a patient with pyelonephritis	CP000247	**4**,**938**,**920 (chromosome**,** circular)**	**87.80%**
**UT189** (UPEC)	O18:K1:H7	B2	Urine, a patient with cystitis	CP000243	**5**,**076**,**615 (chromosome**,** circular)**	**85.66%**
				CP000244	114,230 (plasmid, pUT189)	32.27 and 0%

Footnote: The mapping rates were calculated by using the quality assessment tool for genome assemblies (QUAST). AIEC, adherent-invasive *E. coli*; DAEC, diffusely adherent *E. coli*; EPEC, enteropathogenic *E. coli*; EIEC, enteroinvasive *E. coli*; EHEC/STEC, enterohemorrhagic *E. coli*/Shiga toxin-producing *E. coli*; ETEC; enterotoxigenic *E. coli*; UPEC, uropathogenic *E. coli*.

The sequences of pathobiont strains with adherent-invasive characteristics (AIEC strains of LF82 and NC101) or with diffusely adherent properties (DAEC strains of SK1144 and 2017 C-4173W12) were compared with the genome of LI60C3. The DAEC strains are *E. coli* that harbor afimbrial adhesins encoded by *afa* genes, which were isolated from healthy or diarrheal patients [59–61]. In a sequence alignment with LI60C3, the chromosome of LF82 exhibited the highest mapping rate of 91.00%, while the NC101 strain showed a sequence homology of 86.97% (Table 2). On the other hand, SK1144 and 2017C-4173W12 were matched with 83.93% and 75.53% sequence homology, respectively (Table 2).

The *E. coli* strains 536 and UT189, classified as UPEC, showed sequence homology to LI60C3 with mapping rates of 87.80% and 85.66%, respectively. Other pathogenic *E. coli*, such as E2348/69 (an EPEC strain), CFSAN029787 (an EIEC strain), EDL933 (an EHEC/STEC strain), and H10407 (an ETEC strain), had less similarity with LI603 that ranges from 69.67 to 82.95%. Among the chromosome sequences of pathogenic *E. coli*, the UPEC strains had the highest and the EHEC strains had the lowest genome homology to LI60C3 (Table 2). By comparing with plasmids found in the reference *E. coli* strains, the sequence of pTra in LI60C3 is partially homologous to that of p948 in H10407 (an ETEC strain) with a 41.63% similarity and to that of pUT189 in UT189 (a UPEC strain) with a 32.27% similarity (Table 2). No sequence homology was observed between pTra and the plasmid pLF82 in the AIEC strain LF82 (Table 2).

**Fig. 3:**
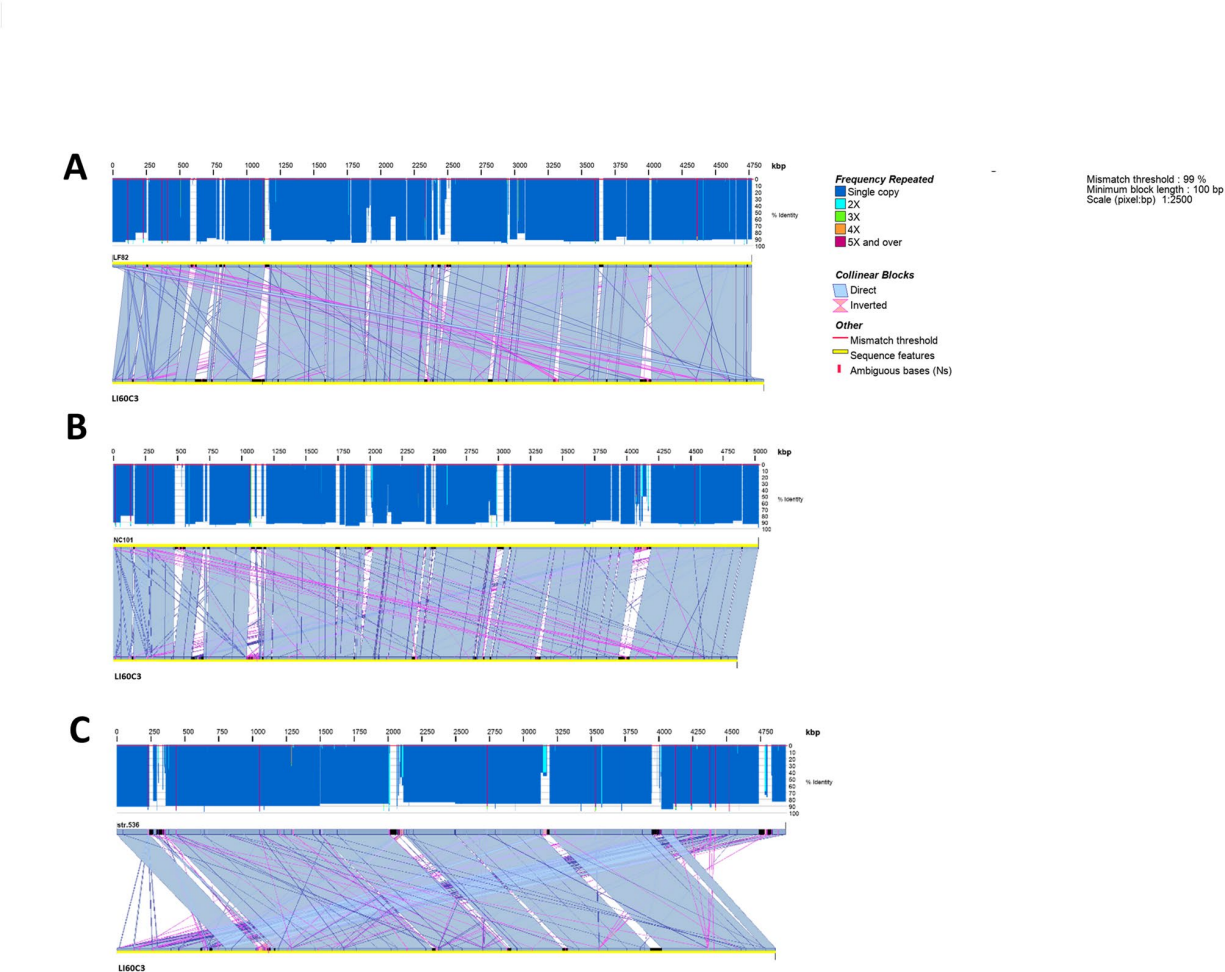
Synteny plots of the LI60C3 genome with other reference strains. High sequence similarity and conserved gene clusters were identified between LI60C3 and other *E. coli* strains, such as the AIEC strains **(A)** LF82 and **(B)** NC101, and **(C)** a UPEC strain 536

The conserved gene clusters identified within the LI60C3 sequence were aligned with the three *E. coli strains* exhibiting the highest mapping accuracy, as depicted in the colinear regions in the synteny plots (Fig. 3). The genome rearrangements are demonstrated in homologous synteny blocks in a pairwise manner between LI60C3 and other *E. coli* strains, such as LF82 (Fig. 3A), NC101 (Fig. 3B), and 536 (Fig. 3C). In summary, the chromosome sequence of LI60C3 displays a high degree of genome homology with AIEC strains (i.e., LF82 and NC101) and UPEC strains (i.e., 536 and UT189).

### Phylogeny and virulence genes of the *E. coli* LI60C3

**Fig. 4:**
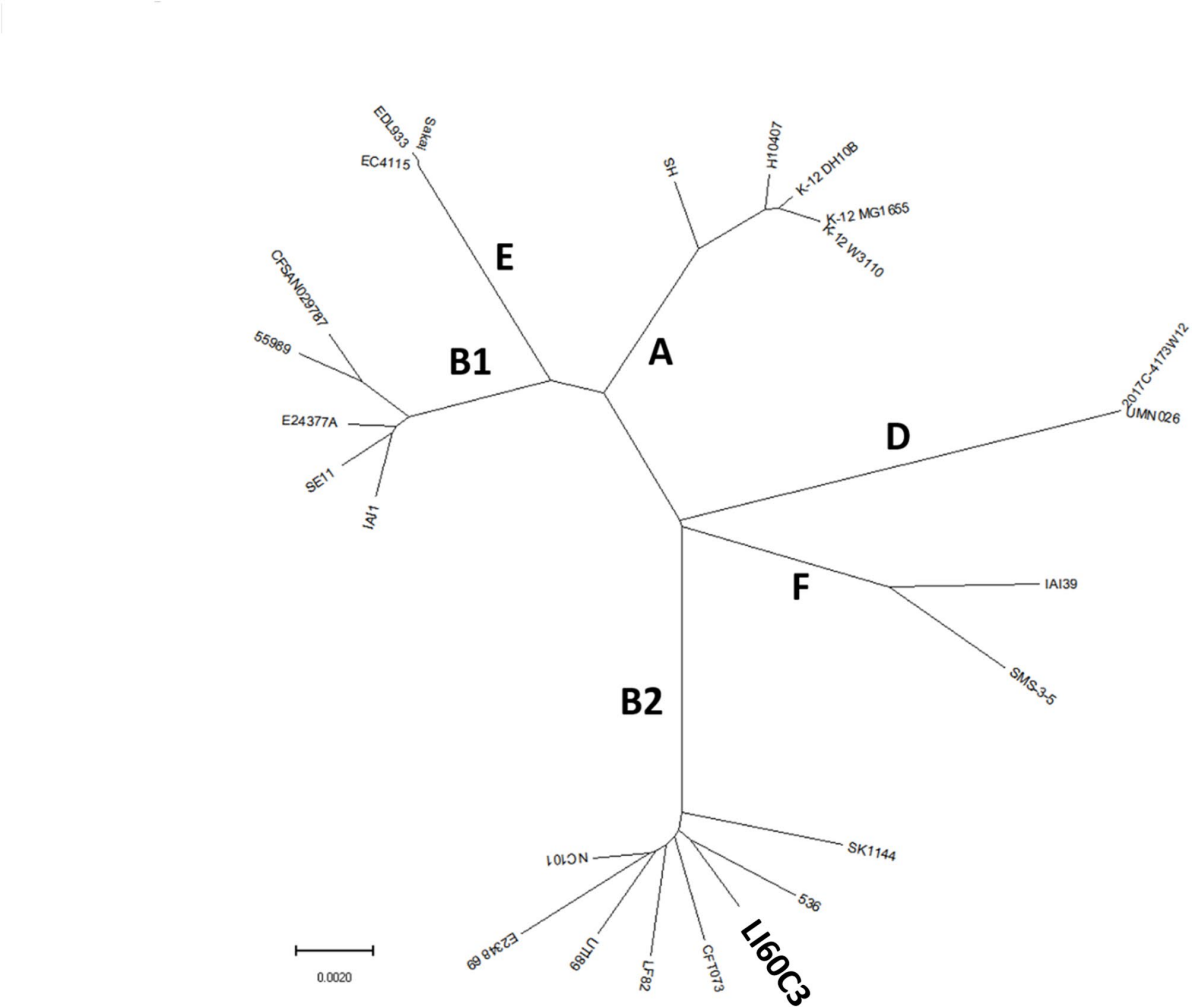
Phylogenetic analysis of *E. coli* strains. The analysis was based on the sequence of seven house-keeping genes (*arcA*,* aroE*,* icd*,* mdh*,* mtlD*,* pgi*, and *rpoS*) of 25 reference strains of *E. coli*, including LI60C3. The major branches are labeled according to the phylogroups (i.e., A, B1, B2, D, E, F), which are written in bold letters. Distance is defined as the fraction of mismatches at aligned positions, with gaps either ignored or counted as mismatches

**Fig. 5:**
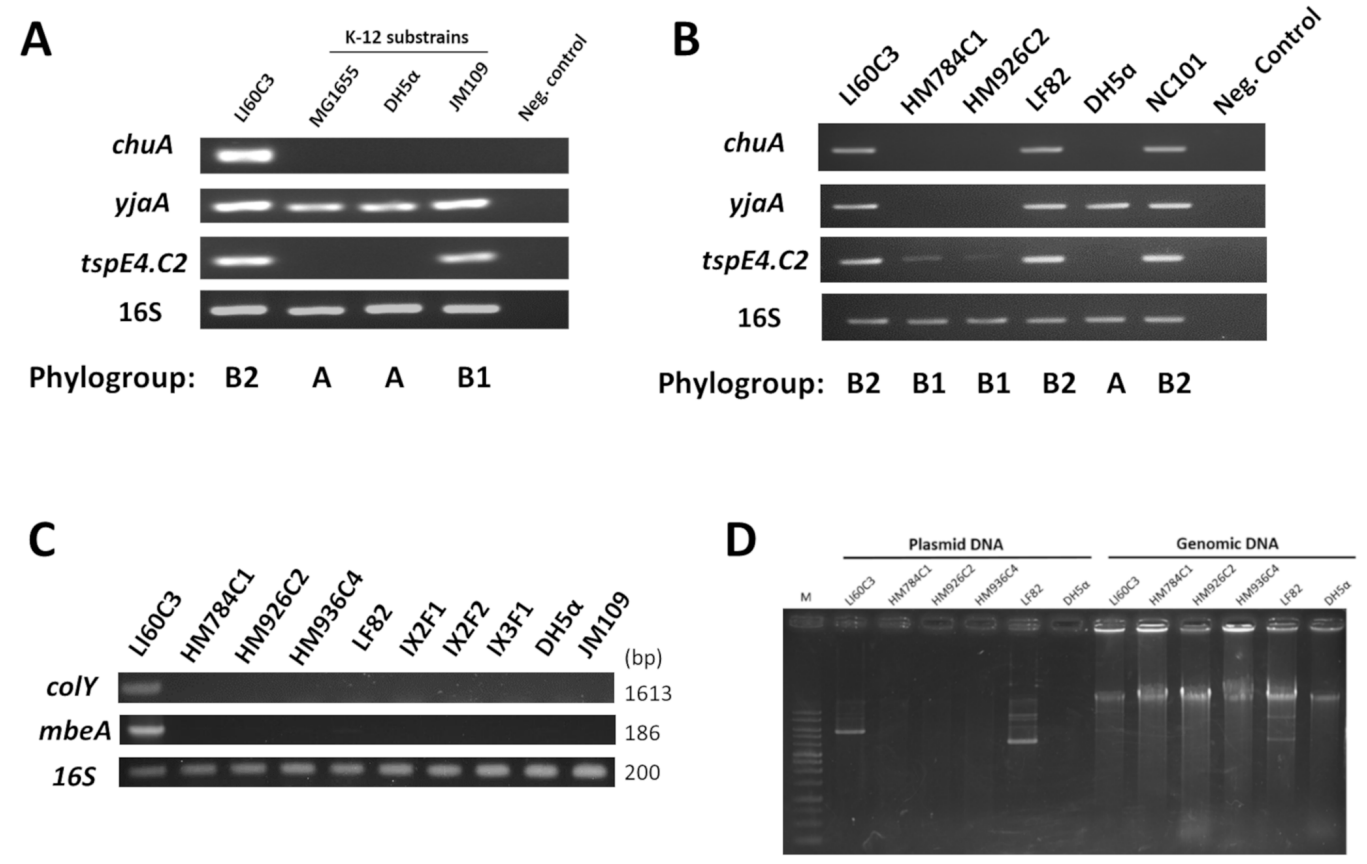
Phylotyping of LI60C3 based on the Clermont method of triplex PCR assay. The phylogroups of *E. coli* were determined using primer pairs targeting three genes (*chuA*, *yjaA*,* and tspE4.C2)*, in accordance with Clermont’s triplex PCR assay. **(A)** Representative gel image showing PCR bands generated from genomic DNA samples of LI60C3 and laboratory *E. coli* strains. The phylogroup of each strain is labeled at the bottom. **(B)** Representative gel image showing the phylogroup of mouse-isolated *E. coli* strains and adherent-invasive LF82 and NC101 strains. **(C)** The plasmid *mbeA* and *colY* genes were found in the genomic DNA samples extracted from LI60C3 but not from other *E. coli* strains. **(D)** Electrophoretic gel images of the extracted plasmid and genomic DNA samples obtained from bacteria. The presence of extrachromosomal plasmids is confirmed in the LI60C3 and LF82 strains but not found in other bacteria. Left: samples eluted from the plasmid extraction kit. Right: samples eluted from the genomic extraction kit

The evolutionary phylogenetic tree was constructed using sequences of seven house-keeping genes *(arcA*,* aroE*,* icd*,* mdh*,* mtlD*,* pgi*, and *rpoS)* from 25 reference strains of *E. coli*, including LI60C3. The phylogeny of LI60C3 is classified as B2, with the closest ancestral root linked to a UPEC strain 536 (Fig. 4). Other *E. coli* strains within the B2 phylogenetic group closely related to LI60C3 included LF82, NC101, E2348/69, UT189, and CFT073 (Fig. 4). The B2 phylogroup of LI60C3 was further validated using Clermont’s triplex PCR assay (Fig. 5A and B). While LF82 and NC101 strains belong to the B2 phylogroup, other intraepithelial *E. coli* isolated from mouse colonocytes, such as HM784C1 and HM926C2, fall within phylogroup B1. In contrast, laboratory K-12 substrains (i.e., MG1655 and DH5α) are classified under phylogroup A, and the strain JM109 belongs to phylogroup B1 (Fig. 5A and B).

Virulence elements and antibiotic-resistance genes in LI60C3 were predicted by sequence alignment using the CARD and VFDB databases. Consistent with our previous antibiogram profile from the antimicrobial susceptibility test, the LI60C3 genome contains genes that confer antibiotic resistance through efflux pumps (Table 3). The LI60C3 genome harbors virulence-encoding genes related to adherence (pilus and fimbriae), invasion, iron uptake, type IV secretion system, and hemolysins (Table 4). Additionally, it was confirmed that the LI60C3 genome lacks both genotoxin and cyclomodulin genes.

**Table 3:** Antibiotic-resistant genes in LI60C3 genome

Resistance mechanism	Gene	Gene Family	Antibiotic Class
antibiotic efflux	*acrA/B*	RND antibiotic efflux pump	fluoroquinolone antibiotic, cephalosporin, glycylcycline, penam, tetracycline antibiotic, rifamycin antibiotic, phenicol antibiotic
	*cpxA*	RND antibiotic efflux pump	aminoglycoside antibiotic, aminocoumarin antibiotic
	*emrY*	MFS antibiotic efflux pump	tetracycline antibiotic
	*evgA*	MFS and RND antibiotic efflux pump	macrolide antibiotic, fluoroquinolone antibiotic, penam, tetracycline
	*emrR*	MFS antibiotic efflux pump	fluoroquinolone antibiotic
	*gadW*	RND antibiotic efflux pump	macrolide antibiotic, fluoroquinolone antibiotic, penam
	*hns*	MFS and RND antibiotic efflux pump	macrolide antibiotic, fluoroquinolone antibiotic, cephalosporin, cephamycin, penam, tetracycline antibiotic
	*kdpE*	kdpDE	aminoglycoside antibiotic
	*leuO*	MFS antibiotic efflux pump	nucleoside antibiotic, disinfecting agents and antiseptics
	*mdfA*	MFS antibiotic efflux pump	tetracycline antibiotic, phenicol antibiotic, disinfecting agents, antiseptics
	*mdtG*	MFS antibiotic efflux pump	phosphonic acid antibiotic
	*mdtH*	MFS antibiotic efflux pump	fluoroquinolone antibiotic
	*mdtP*	MFS antibiotic efflux pump	nucleoside antibiotic, disinfecting agents, and antiseptics
	*mdtO*	MFS antibiotic efflux pump	nucleoside antibiotic, disinfecting agents, and antiseptics
	*mdtN*	MFS antibiotic efflux pump	nucleoside antibiotic, disinfecting agents, and antiseptics
	*msbA*	ABC antibiotic efflux pump	nitroimidazole antibiotic
	*marA*	RND antibiotic efflux pump, General Bacterial Porin with reduced permeability to beta-lactams	fluoroquinolone antibiotic, monobactam, carbapenem, cephalosporin, glycylcycline, cephamycin, penam, tetracycline antibiotic, rifamycin antibiotic, phenicol antibiotic, penem, disinfecting agents, antiseptics
antibiotic target alteration	*eptA*	pmr phosphoethanolamine transferase	peptide antibiotic
	*vanG*	glycopeptide resistance gene cluster, Van ligase	glycopeptide antibiotic
antibiotic inactivation	*EC-5*	EC beta-lactamase	cephalosporin

Footnote: Resistance genes were predicted by the Resistance Gene Identifier (RGI) (v.6.0.3) which performed homolog detection using DIAMOND in Comprehensive Antibiotic Resistance Database (CARD) (v.3.3.0). ABC, ATP-binding cassette; MFS, major facilitator superfamily; RND, resistance-nodulation-cell division

**Table 4:** Virulence-encoding genes in LI60C3 genome

Virulence Class	Virulence Factors	Gene	Phylogroup B2								Phylogroup A	
			LI603	LF82	N101	536	UT189	CFT073	SK1144	E2348/69	MG1655	W3110
Adherence	E. coli common pilus	*ecp* *A/B/C/D/E*	+	+	+	+	+	+	+	+	+	+
	Hemorrhagic E. coli pilus	*hcp* *A/B/C*	+	+	+	+	+	+	+	+	+	+
	P fimbriae	*pap* *C/D/H*	+	-	-	+	+	+	+	-	-	-
	Type 1 fimbriae	*fim* *A/C/D/E/F/G/H/I*	+	+	+	+	+	+	+	+	+	+
Autotransporter	Contact-dependent inhibition CDI system	*cdiA*	+	-	+	+	+	+	-	-	-	-
	Vacuolating autotransporter gene	*vat*	+	+	+	+	-	+	-	-	-	-
Invasion	Invasion of brain endothelial cells	*ibe* *B/C*	+	+	+	+	+	+	+	+	+	+
Iron uptake	Heme uptake	*chu* *A/X/T/U/W/X/Y*	+	+	+	+	+	+	+	+	-	-
	Iron/manganese transport	*sit* *A/B/C/D*	+	+	+	+	+	+	-	-	-	-
	Yersiniabactin siderophore	*ybt* *A/E/P/Q/S/T/U/X*	+	+	+	+	+	+	-	-	-	-
Secretion system	Non-LEE encoded TTSS effectors	*espL1* *espR1*	+	+	+	-	+	+	- ^a^	- ^a^	+	+
	SCI-I T6SS	*Undetermined*	+	+	+	+	+	+	-	-	-	-
Toxin	Hemolysin/cytolysin A	*hlyE/clyA*	+	+	+	-	+	-	-	-	+	+
Antimicrobial activity	Phenazine biosynthesis	*phzB2* *phzF2*	+	-	-	-	-	-	-	-	-	-
Serum resistance	LPS rfb locus	Undetermined	+	-	-	-	-	-	-	-	-	-

Footnote: Virulence factor-encoding genes were predicted based on sequence alignment using the virulence factor database (VFDB). LEE, locus of enterocyte effacement; TTSS, Type III secretion systems; T6SS, type VI secretion system; CDI, contact-dependent growth inhibition

^a^ Contains other *esp* or *nle* genes encoding for the non-LEE encoded TTSS effectors

### Two extrachromosomal plasmids were detected in the LI60C3 genome

The presence of two extrachromosomal plasmids in LI60C3 was revealed by WGS analysis, which were derived from contigs assembled from PacBio sequencing. The larger plasmid named pTra (81,809 bases) contains *tra* genes that encode for transfer proteins involved in plasmid DNA transfer during bacterial conjugation, such as pili synthesis and assembly, and helicase activity. Several *tra* genes are annotated in the 82-kb plasmid sequence, including *traI*,* traD*,* traS*,* traQ*,* traC*,* traV*,* traA*,* traY*,* traJ*,* and traM*. In the *tra* operons, *traA* encodes the precursor of the pilus subunit propilin; *traQ* gene products facilitate TraA maturation; *traI* gene encodes for helicase with DNA unwinding activity, and *traY*, *traJ*, and *traM* are involved in the autoregulatory pathway to induce DNA bending and stimulate nicking [62]. We used the basic local alignment search tool (BLAST) in NCBI to search for plasmids in public databases with homology to pTra. The NCBI blasting indicated that several plasmids in commensal *E. coli* strains exhibited a 99.75% genome identity with a query coverage of 57% when compared to the pTra sequence (Table 5).

**Table 5:** Genome comparison of LI60C3 pTra to other plasmids

Plasmid Name	GenBank Accession No.	Genome Size (bp)	Query Coverage (%)	Percent Identity (%)	Total Score
Escherichia coli strain MG1655 K12 plasmid F-Tn10	MK492260	108,314	57	99.75	85,004
Escherichia coli strain Stbl4 plasmid pF	CP076044	98,268	57	99.75	84,952
Escherichia coli K-12 plasmid F DNA	AP001918	99,159	57	99.75	84,952
Escherichia coli strain NEB5-alpha_F’Iq plasmid F’Iq	CP053608	242,042	57	99.75	84,991
Escherichia coli strain NEB_Turbo plasmid F’	CP053606	231,547	57	99.75	84,996

Footnote: The complete sequence of plasmids was compared using NCBI blasting. Plasmids with a query coverage of less than 56% are not shown, as this is set as the main threshold

The smaller plasmid named pCoMb (8,556 bases) encompasses several genes encoding for colicin Y (colY), colicin lysis, colicin immunity protein, DNA relaxase/mobilization protein (mbe) A and mbeC, regulatory protein Rop, and entry exclusion protein. The annotated genes in pCoMb were also confirmed by Sanger sequencing on the plasmid DNA samples extracted from LI60C3 using a commercially available Mini Plasmid kit. The NCBI blasting showed that pCoMb closely matched the plasmid sequences found in *E. coli* strains and other pathogenic bacteria. These include the plasmids reported in *E. coli* strain NGF1 (Accession no. CP016009) and O25b: H4-ST131 strain 2017 (Accession no. CP051617) with a query coverage of 100% and a percent identity over 99%, and the plasmid pHS08-175-2-52445 bp-repAIncN in *Enterobacter hormaechei* (Accession no. MT010563) with a query coverage of 98% and a percent identity of 96%, and unnamed plasmids in *Klebsiella pneumoniae* (Accession no. CP054989) and *Shigella flexneri* (Accession no. CP055169) with a query coverage of 51% and a percent identity of 97% (Table 6).

**Table 6:** Genome comparison of pCoMb to other plasmids

Plasmid Name	GenBank Accession No.	Genome Size (bp)	Query Coverage (%)	Percent Identity (%)	Total Score
Escherichia coli strain NGF1 plasmid pNGF1_pCol_let_like	CP016009	8,556	100	100	15,796
Escherichia coli O25b: H4-ST131 strain 2017_APHA plasmid pAPHA_2017_2	CP051617	8,554	100	99.69	15,667
Enterobacter hormaechei subsp. steigerwaltii strain 08-175 plasmid pHS08-175-2-52445 bp-repAIncN	MT010563	52,445	98	95.88	11,882
Escherichia coli strain SCU-101 plasmid pSCU-101-1	CP051850	7,472	79	98.51	12,211
Escherichia coli pCol-let plasmid	AF1967335	5,847	56	99.35	8675
Klebsiella pneumoniae strain STIN_90 plasmid unnamed5	CP054989	6,817	51	97.04	6751
Shigella flexneri strain STEFF_24 plasmid unnamed2	CP055169	6,817	51	97.04	6741

Footnote: The complete sequence of plasmids was compared using NCBI blasting. Plasmids with a query coverage of less than 50% are not shown, as this is set as the main threshold

Furthermore, the plasmid DNA was extracted from different bacterial strains for PCR analysis. Among the isolated *E. coli* strains from mice, only LI60C3 contained a plasmid with the *mbeA* and *colY* genes; the other strains isolated from mouse colonocytes (HM784C1, HM926C2, and HM936C4) and fecal bacteria (IX2F1, IX2F2, and IX3F1) did not (Fig. 5C). Moreover, nonpathogenic laboratory strains of DH5α and JM109 also exhibited the absence of plasmids containing *mbeA* and *colY* genes (Fig. 5C). Since the presence of a plasmid pLF82 (Accession no. CU638872) was reported in the LF82 genome, we stained the plasmid DNA with a bioluminescent dye on the electrophoretic gel. Multiple bands were shown on the gel, including a sharp band at the 10-kb site, which could be pLF82 (Fig. 5D). According to the NCBI blasting results, the plasmids in LF82 had no sequence homology with pTra and pCoMb in LI60C3.

### Unique gene clusters named Epm and Phz Islands in the LI60C3 chromosome

Unique gene clusters were identified in the LI60C3 genome by comparing the whole genome with reference *E. coli* strains (Table 7). One of the two unique gene clusters in the LI60C3 genome was designated as the Epm (epimerase) island, which included one copy of genes encoding for capsule biosynthesis protein (*capA*), UDP-N-acetylglucosamine 4-epimerase *(wbgU)*, and dTDP-4-dehydrorhamnose 3,5-epimerase *(rmlC)* (Table 7). The structures of capsules (K-antigen), lipopolysaccharides (LPS) (O-antigen), flagella (H-antigen), and fimbriae (F-antigen) are essential virulence determinants for serotyping *E. coli* in relation to clinical infections [63]. The *capA* gene encodes for subtypes of capsular polysaccharides that enable the bacteria to evade or counteract the innate host defense during the early phase of infection. The *wbgU* and *rmlC* genes encode for epimerases which are enzymes acting on carbohydrates and are involved in LPS synthesis. In summary, the Epm genes account for synthesizing the particular capsular and LPS subtypes of the bacteria.

**Table 7:** Specific gene clusters in LI60C3 genome unmatched to reference strains

Gene	Protein	start	stop	Strand	bp
*wbgU*	UDP-N-acetylglucosamine 4-epimerase	2,932,700	2,933,704	+	1004
*rmlC*	dTDP-4-dehydrorhamnose 3,5-epimerase	2,936,733	2,937,275	-	542
*capA*	Capsule biosynthesis protein CapA	2,938,144	2,939,148	-	1004
*dhbF*	Dimodular nonribosomal peptide synthase	3,955,229	3,956,998	-	1769
*phzG*	Phenazine biosynthesis protein PhzG	3,960,417	3,961,052	-	635
*phzF*	Trans-2,3-dihydro-3-hydroxyanthranilate isomerase	3,961,066	3,961,911	-	845
*phzD*	Phenazine biosynthesis protein PhzD	3,964,102	3,964,722	-	620
*phzB1*	Phenazine biosynthesis protein PhzB1	3,964,798	3,965,256	-	458
*ehpR*	Phenazine antibiotic resistance protein EhpR	3,966,190	3,966,576	-	386
*yadA*	Adhesin YadA	3,995,109	3,997,334	-	2225

Footnote: Sequence alignment was determined by NCBI blasting. The nucleotide locations and lengths of the gene in LI60C3 that did not match any reference *E. coli* were summarized

The second unique gene cluster, named the Phz (phenazine) island, consists of single copies of the *dhbF gene* and a series of genes that encode proteins involved in phenazine biosynthesis (Table 7). The *dhbF* gene encodes for a dimodular nonribosomal peptide synthase. The *phzG*,* phzF*,* phzD*,* phzB1*, and * ehpR* genes respectively encode for phenazine biosynthesis protein G, F, D, and B1, and the phenazine antibiotic resistance protein. Phenazines are bacterial secondary metabolites that are oxidases, yielding superoxide and peroxide ions and hydroxyl radicals. These reactions may confer competitive growth advantages for microbes [64].

### Mouse and human CRC specimens exhibit higher levels of LI60C3 genetic signatures compared to healthy mucosal samples

**Fig. 6:**
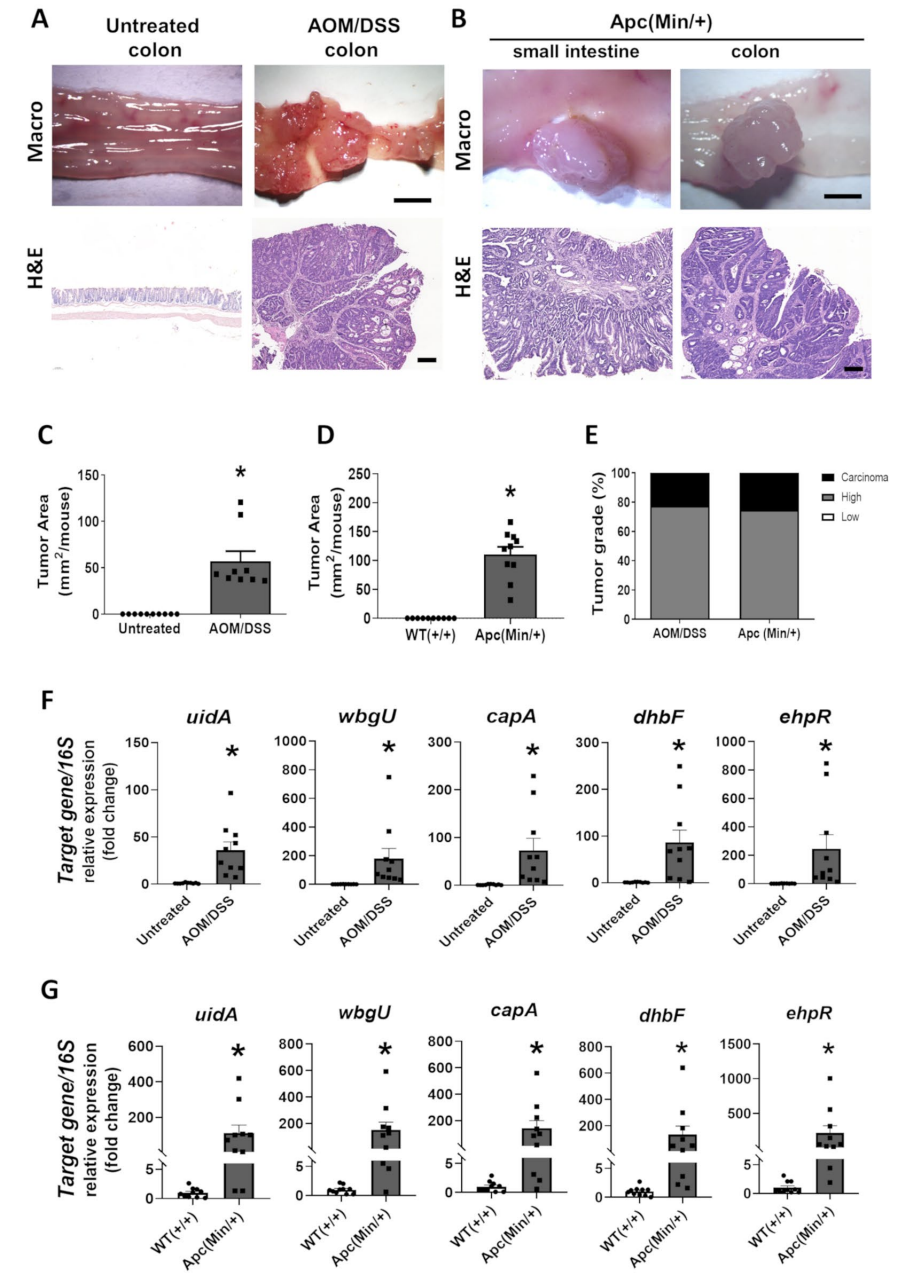
LI60C3-specific genes were detected in mouse tumors but are absent in the intestinal tissues of healthy control mice. Unique gene clusters of LI60C3 are designated as the Epm island (*wbgU* and *capA* genes) and the Phz island (*dhbF* and *ephR* genes). The levels of LI60C3 biomarkers were examined in mouse tumors induced by chemicals and genetic mutation. **(A** and **B)** Representative images of intestinal tumors in the AOM/DSS and Apc(Min/+) mice. Bar: 2 mm (macroscopic images) and 200 μm (histological images with H&E staining). **(C)** Tumor area in the untreated and AOM/DSS mice. **(D)** Tumor area in the WT(+/+) and Apc(Min/+) mice. Data are expressed as mean ± SEM (*N* = 8–10/group). Each dot represents the data of one mouse. *P < 0.05 *vs.* untreated or WT(+/+) mice, as calculated by Student’s *t* test. **(E)** The percentages of tumors with low- and high-grade dysplasia, and carcinoma in the AOM/DSS and Apc(Min/+) mice. **(F** and **G)** Quantitative PCR analysis of *uidA*,* wbgU*,* capA*,* dhbF*, and *ephR* levels in the tumor tissues of AOM/DSS and Apc(Min/+) mice. Data are expressed as mean ± SEM (*N* = 8–10/group). Each dot represents the data of one mouse. *P < 0.05 *vs.* untreated or WT(+/+) mice, as calculated by Mann-Whitney U test

The presence of LI60C3 genetic signatures was examined in mouse tumor tissues from two established models, including chemically induced AOM/DSS mice and genetic mutant Apc(Min/+) mice, and compared to the intestinal samples from their corresponding controls without tumors (Fig. 6A-E). The genomic DNA was extracted from mouse intestinal tissues and analyzed by qPCR. The relative expression levels of the *E. coli*-specific *uidA* gene, normalized to the bacterial *16S rRNA*, were higher in the AOM/DSS mice than in the untreated mice, indicating an abundance of *E. coli* in tumor tissues. The AOM/DSS tumor samples exhibited significantly increased levels of LI60C3-specific genes (i.e., *wbgU*,* capA*, *dhbF*, and *ephR*) compared to colonic tissues of untreated mice (Fig. 6F). Similarly, elevated levels of the *E. coli uidA* gene and LI60C3-specific genes were detected in tumor samples of Apc(Min/+) mice compared with normal tissues of WT(+/+) littermates (Fig. 6GF).

**Fig. 7:**
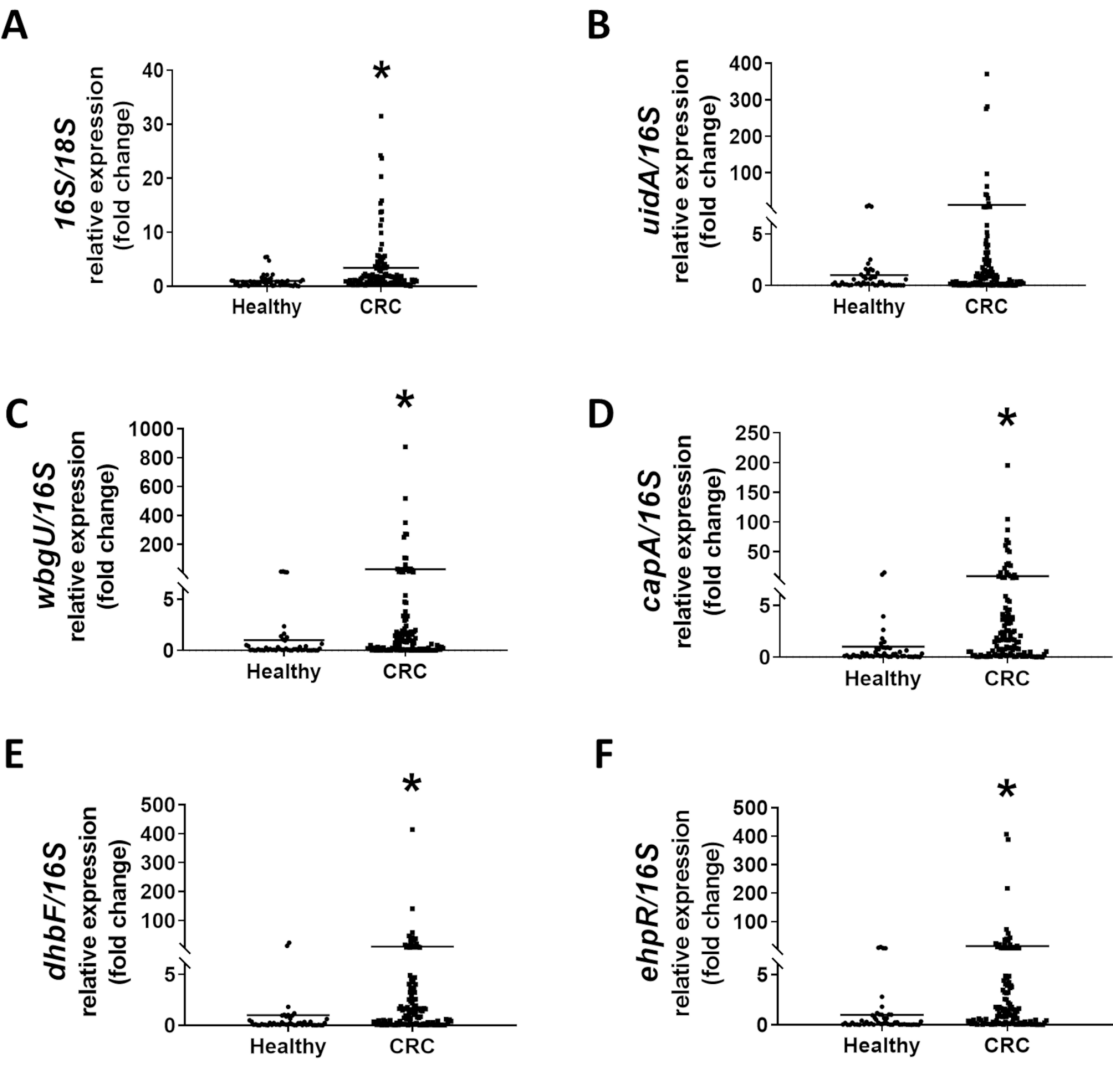
Human CRC specimens exhibit higher levels of LI60C3 genetic signatures compared to healthy mucosal samples. Colonic specimens from healthy individuals (*N* = 49) and CRC patients (*N* = 114) were analyzed for bacterial genes. **(A)** The levels of the universal bacterial marker (*16S rRNA* gene) in CRC specimens were higher than those in healthy mucosal samples. The expression of the bacterial *16S rRNA* gene was normalized to the levels of the human *18S rRNA* gene. **(B)** The amount of the *E. coli*-specific *uidA* gene in CRC specimens showed an increasing trend compared to healthy mucosal samples. **(C-F)** The levels of LI60C3-specific genes (*wbgU*, c*apA*,* dhbF*, and *ephR)* were significantly elevated in CRC specimens when compared to healthy mucosal samples. The expression of LI60C3-specific genes was normalized to the levels of *16S rRNA*. Each dot represents the data of one sample, and the line indicates the mean value of the group. *P < 0.05 vs. Healthy, as calculated by Mann-Whitney U test

Lastly, the expression of LI60C3-specific genes was validated in the colonic specimens of healthy individuals and patients with CRC. The human tissue RNA was reverse-transcribed to cDNA samples for qPCR analysis. A 3.4-fold higher bacterial amount was observed in the CRC specimens than in the healthy mucosal samples, as identified by a higher ratio of *16S* to *18S rRNA* genes in tumors (Fig. 7A). The *E. coli*-specific *uidA* gene levels were slightly increased in the CRC specimens compared with the healthy mucosal samples, albeit without statistical significance. Moreover, the abundance of LI60C3-specific genes (i.e., *wbgU*,* capA*,* dhbF*, and *ephR)* was 27, 9, 9, and 14 times higher in the CRC specimens than the healthy mucosal samples, respectively (Fig. 7B-F). When the data were stratified into the four stages of CRC, elevated amounts of *capA* were noted at tumor stage II and III, and an increase in the *ephR* level was found at tumor stage III relative to healthy individuals (Table 8).

**Table 8:** The LI60C3-specific gene levels in healthy mucosal samples and CRC specimens

Gene /16S (mean ± SEM)	Healthy (*N* = 49)	CRC (*N* = 114)		*P* value
***16 S***	1.0 ± 0.2	3.4 ± 0.5*		***P*** **< 0.0001**
		I II III IV	3.2 ± 1.3 2.6 ± 0.6* 4.2 ± 1.1* 4.0 ± 1.6	*P* = 0.097 ***P*** **= 0.009** ***P*** **= 0.007** *P* = 0.069
***uidA***	1.0 ± 0.3	11.9 ± 4.8*		***P*** **= 0.028**
		I II III IV	1.4 ± 0.5 23.2 ± 11.9 11.6 ± 8.3 0.6 ± 10.2	*P* = 0.467 *P* = 0.070 *P* = 0.212 *P* = 0.332
***wbgU***	1.0 ± 0.4	27.4 ± 10.1 *		***P*** **= 0.010**
		I II III IV	29.3 ± 25.8 36.4 ± 23.3 22.0 ± 13.1 17.7 ± 12.0	*P* = 0.287 *P* = 0.137 *P* = 0.120 *P* = 0.181
***capA***	1.0 ± 0.4	9.0 ± 2.3 *		***P*** **= 0.001**
		I II III IV	4.0 ± 2.6 16.0 ± 6.1 * 7.8 ± 2.9 * 2.8 ± 1.4	*P* = 0.260 *P* = **0.018** *P* = **0.028** *P* = 0.214
***dhbF***	1.0 ± 0.5	9.0 ± 3.9 *		***P*** **= 0.046**
		I II III IV	1.8 ± 0.5 16.9 ± 11.2 9.9 ± 4.5 1.2 ± 0.4	*P* = 0.275 *P* = 0.166 *P* = 0.059 *P* = 0.796
***ehpR***	1.0 ± 0.3	14.0 ± 5.4 *		***P*** **= 0.017**
		I II III IV	3.6 ± 1.9 26.5 ± 14.9 7.8 ± 2.8 * 11.8 ± 9.8	*P* = 0.200 *P* = 0.096 *P* = **0.020** *P* = 0.286

Footnote: Colonic specimens were collected from 49 healthy individuals (age interval: 33–78 years) and 114 patients with colorectal carcinoma (CRC) (age interval: 26–88 years). The number of patients at tumor stages I, II, III, and IV were 20, 39, 33, and 22, respectively. Tissue RNA was reverse-transcribed and analyzed by qPCR. Data presented as fold change of relative expression (mean ± SEM). * *P* < 0.05 vs. healthy by Student’s *t* test

## Discussion

Our previous work demonstrated microbial virulence emergence during a key phase of colon cancer initiation, and an invasive *E. coli* LI60C3 isolated from mouse colonocytes was identified with tumorigenic and colitogenic ability [21, 22]. To clarify the genetic characteristics of this pathobiont, the bacterial genomic DNA was sent for whole-genome sequencing. When compared to other *E. coli* strains with similar invasive capabilities, LI60C3 exhibited a greater ability to promote epithelial proliferation and increase tumor burden in mice. The assembly of long reads revealed a circular chromosome and two extrachromosomal plasmids in LI60C3. Unique gene clusters were identified in the chromosome of LI60C3, with higher levels observed in mouse tumors and human colorectal cancer specimens compared to healthy mucosal samples. Having reported the first complete genome of this bacterial species, the information will serve as a foundation for comparative genome analysis aimed at identifying oncogenic signatures.

We conducted a genomic comparison of the protumoral *E. coli* LI60C3 strain to commensal K-12 strains, AIEC pathobiont strains, and pathogenic strains responsible for intestinal and extraintestinal diseases. The highest mapping rates were observed when comparing the LI60C3 genome to those of AIEC strains, such as LF82 and NC101 (> 90%) followed by UPEC strain*s* (> 88%). The LI60C3 strain expressed the *fim* operon encoding Type 1 fimbriae, *pap* genes for P fimbriae, and *lpfA* gene encoding long polar fimbriae for adherence, but did not contain the *afa* gene. The LF82 strain, with Type 1 fimbriae and colitogenic ability, displays the highest genome homology to LI60C3 but has no effect on mouse tumor growth. The NC101 strain contains the *fim* operon encoding Type 1 fimbriae and *afa* genes encoding afimbrial adhesins; it also produces genotoxic colibactin to cause DNA damage and promote tumor growth in murine models. Although there is a high degree of sequence homology between the tumorigenic *E. coli* strains LI60C3 and NC101, it is noteworthy that the polyketide synthase *(pks)* pathogenicity island is absent in the LI60C3 genome. These findings suggest that LI60C3 may promote tumorigenesis through different mechanisms compared to the colibactin-producing NC101. The UPEC strains, such as 536 and UT189, expressed the *fim* operon and *pap* genes for adherence, similar to LI60C3. The sequence homology between LI60C3 and commensal *E. coli* is approximately 80%, which is higher than its genome similarity to pathogenic EIEC, EHEC, or ETEC strains, ranging from 70 to 78%. The commensal laboratory *E. coli* strains, MG1655 and W3110, are sub-strains of K-12 isolated from human feces [65, 66]. The MG1655 strain was chosen as a reference nonpathogenic *E. coli* since it has been maintained in the laboratory with minimal genetic manipulation and has only been cured of the temperate bacteriophages [65, 66]. Other laboratory bacteria derived from the ancestral K-12 strain, such as DH5α and JM109, are not suitable for genome comparison. The DH5α and JM109 strains, used as competent cells with maximized plasmid transformation efficiency, were engineered for *recA1* and *endA1* gene mutations to inactivate homologous recombination and intracellular endonuclease [67, 68]. In summary, LI60C3 exhibited high sequence homology with AIEC strains, and its genome showed greater ancestral similarity to commensal *E. coli* than to pathogenic strains.

Unique gene clusters were identified in the LI60C3 chromosome sequence, referred to as Epm and Phz islands, which could serve as specific markers of the pathobiont. One of the gene clusters, named Phz (phenazine) island, contains multiple genes *phzG*,* phzF*,* phzD*,* phzB1*, and *ehpR* encoding for phenazine families. Phenazines are bacterial secondary metabolites that serve as electron shuttles to alternate terminal acceptors, modify cellular redox states, act as cell signals that regulate gene expression patterns, contribute to biofilm formation and architecture, and enhance bacterial survival. The Epm (epimerase) island, encompassing the *capA*,* wbgU*, and *rmlC* genes, is involved in synthesizing bacterial cell surface structures. Microbial capsules (K-antigen), lipopolysaccharides (O-antigen), flagella (H-antigen), and fimbriae (F-antigen) are essential virulence determinants for serotyping *E. coli* in clinical infections [63]. The *capA* gene encodes for capsular polysaccharides that enable the bacteria to evade or counteract the innate host defense during the early phase of infection. The capsules could also act as receptors for some bacteriophages containing capsule depolymerase while preventing infection of other phages [69]. Moreover, the *wbgU* and *rmlC* genes encoding for epimerases (UDP-N-acetylglucosamine 4-epimerase and dTDP-4-dehydrorhamnose 3,5-epimerase) are involved in the synthesis of the O side-chain of LPS. A few reports have classified UPEC strains in urinary tract and bloodstream infections with O: K:H serotypes [70, 71]. Here, we identified a high sequence similarity of LI60C3 with UPEC strains, i.e., 536 (88%) and UTI89 (86%), which possess the K1 and K2 antigen types of capsules [71]. In summary, the Epm island on the LI60C3 genome is responsible for synthesizing particular capsular and LPS subtypes. These genes could be useful biomarkers to differentiate the existence of pathobionts from other commensal *E. coli* in fecal samples.

As we demonstrated that LI60C3 promoted neoplasia formation in animal models, the validation of pathobiont emergence during the course of tumorigenesis would support their commensal roots in the intestinal microbiota. High levels of LI60C3-specific genes were exhibited in two mouse tumor models and human CRC specimens compared with their healthy counterparts. Previous reports showed a high abundance of *E. coli* in intestinal polyps and tumor tissues of patients with FAP and CRC [15–20]; however, microbial genetic signatures to distinguish between commensals and pathobionts are lacking. The presence of pathobiont markers in biopsy samples may confer prognostic value for predicting tumor advancement. Moreover, detection of serum immunoglobulins targeting bacterial outer membrane antigens, such as those encoded by the *capA* gene, may signal microbiota-associated tumor progression. Such markers could indicate malignant transformation or metastasis, thereby aiding in more accurate assessments of patient outcomes and guiding treatment decisions. Overall, genetic signatures of the tumorigenic LI60C3 strain may be used as cancer predictors.

LI60C3 expresses virulence elements that are also present in known AIEC strains (e.g., LF82 and NC101), including Type 1 and P fimbriae, siderophores, non-LEE encoded TTSS effectors, and hemolysin/cytolysin A. The virulent roles of FimH variants, FadA, cyclomodulins, and colibactin were implicated in colitis and tumorigenesis. Point mutations in the *fimH* gene confer AIEC bacteria a significantly higher ability to adhere to CEACAM-expressing T84 intestinal epithelial cells and to worsen gut inflammation in the infected CEABAC10 transgenic mice expressing human CEACAM receptors [72]. Our previous study has demonstrated that deletion of the *fimH* or *htrA* gene partially inhibited the tumorigenic and proliferation-inducing abilities of LI60C3 ^21^. However, these cannot be considered pathogenic factors for oncogenicity since all *E. coli* harbor Type 1 fimbriae for adhesion and htrA proteases to enhance bacterial survival and replication under stress conditions [73]. Other reports indicate that the FadA adhesin/invasin conserved in *Fusobacterium nucleatum* (an oral anaerobic bacterium with tumorigenic capability) binds to E-cadherins on epithelial cells and activates Wnt/beta-catenin pathways [74, 75]. The presence of operons encoding Type 1 and P fimbriae and long polar fimbriae, and the *fadA* gene (nt. 11436–115699) was observed in the LI60C3 genome. Bacteria-derived cyclomodulins and genotoxins cause DNA damage and cell cycle arrest, which were implicated in oncogenesis [76, 77]. Colibactins, encoded by the *Clb* pathogenicity island, promoted cell senescence and tumor growth in mice [26, 30, 31]. Consistent with our previous qPCR data, genes encoding for cyclomodulin-related toxin (*crt*), cytolethal distending toxin (*cdt*), cytotoxic necrotizing factor (*cnf*), and colibactin *(clb)* or polyketide synthase *(pks)* were absent in the LI60C3 genome. Since none of the aforementioned genotoxins were found, whether the Epm and Phz islands are oncogenic warrants further investigation. Moreover, intestinal inflammation caused by microbial invasion could also be considered as one of the indirect mechanisms of LI60C3-associated tumorigenesis.

Other possible virulence factors in the LI60C3 genome that fit in the pathogenicity frameworks may be encoded by genes on the extrachromosomal plasmids. Bacteria utilize plasmids for horizontal gene transfer through structures known as sex pili, which can significantly enhance their survival by enabling the sharing of beneficial traits, such as toxin production and invasive capabilities [78]. This process is particularly prevalent among bacteria residing in various ecosystems, including the intestinal microflora. The evolutionary importance of virulence acquisition through plasmid mobility is essential for microbial adaptation and resilience in diverse environments, and may contribute to the oncogenic ability of pathobionts. Based on the WGS results, the larger plasmid pTra encompassing *tra* operons resembles many fertility-like plasmids in commensal *E. coli* [62]. The smaller plasmid pCoMb harbors two key proteins, MbeA and ColY, and shares sequence homology with plasmids of pathogenic bacteria such as *Enterobacter hormaechei*, *Klebsiella pneumoniae*, and *Shigella flexneri.* Colicin is one type of bacteriocin produced by *E. coli* for intra-species competition, by binding to outer membrane proteins (OMPs) and being transported into other bacteria where it exerts cytotoxic effects. These effects include depolarizing the cytoplasmic membrane and executing DNase and RNase activities, which can damage the genetic material of the target bacteria [17, 79]. The *mbeA* gene encodes for DNA relaxase, a single-strand DNA transesterase enzyme produced by bacteria. This enzyme is capable of site-specific nicks in double-stranded DNA. The relaxase subclass Mbe protein nicks at the origin of transfer sequence to initiate the transfer of a piece of DNA during bacterial conjugation [80]. Notably, colicin and mobilization genes identified in the pCoMb are also found in the chromosome of LI60C3, suggesting plasmid insertion into the bacterial genome. The oncogenic potential of pathobionts as a consequence of plasmid mobility through bacterial conjugation needs further investigation.

By keeping the multifactorial nature of oncogenesis in mind, there are several limitations of the observational microbiome studies and experimental pathobiont models. First, small sample sizes in microbiome studies can lead to unreliable results due to increased variability and reduced statistical power. The microbiota composition only represents the microbial factors for disease pathogenesis in a subset of patients. Second, fecal microbiota does not reflect the complete population of intestinal microorganisms, and comparison of fecal composition to the microbiota profiles in mucosal compartments and tumor regions may provide a more comprehensive view of gut ecosystem alteration. Third, longitudinal studies on the transition of resident microbes from beneficial commensals to pathogenic bacteria are scarce in the literature. This gap in research hinders our understanding of how microbial communities evolve in response to stressors or upon genetic material transfer through plasmids. Fourth, bacterial strain heterogeneity and variation of virulence elements may account for differences in tumor outcomes. As live microbes divide rapidly and undergo significant changes in their gene makeup compared to mammalian cells, this characteristic could either amplify or obscure the role of microbial factors in the development of host diseases at later stages. Fifth, there is insufficient mechanistic studies of the oncogenic properties of virulence factors. Functional knockouts to validate bacterial gene function for tumorigenesis are still lacking at this stage. It should be cautioned that deletion of specific virulent elements in *E. coli* can result in a reduction of the proliferative response and tumor load; however, this does not mean that the virulence factors per se are oncogenic. For example, the fimbriae system is important for bacteria to adhere to the host, and therefore, the functional impairment of these mutants is likely due to the defect in adhesion, rather than their direct involvement in epithelial proliferation and tumorigenesis.

In conclusion, the genomic characterization of the tumorigenic pathobiont LI60C3 is pivotal in identifying microbial biomarkers for CRC screening. The genome sequence of LI60C3 presents new opportunities for research aimed at differentiating commensal-derived pathobionts from harmless symbionts within the microbiota. This phenotypic and genetic assessment of commensal-derived pathobionts will enhance our understanding and contribute to the advancement of precision interventions targeting the microbiome.

## Supplementary Information

Below is the link to the electronic supplementary material.

Supplementary Material 1

## Data Availability

The whole genome of the bacteria was deposited in GenBank under the accession numbers CP136900, CP136901, and CP136902.

## Abbreviations

AIEC: Adherent-invasive E. coli
AOM: Azoxymethane
Apc: Adenomatous polyposis coli
BLAST: Basic local alignment search tool
CARD: Comprehensive Antibiotic Resistance Database
CDS: Coding sequences
cdt: Cytolethal distending toxin
Clb: Colibactin
CLR: Continuous long-read
colY: colicin Y
cnf: Cytotoxic necrotizing factor
crt: Cyclomodulin-related toxin
CRC: Colorectal cancer
DSS: Dextran sodium sulfate
DAEC: Diffusely adherent E. coli
EAEC: Enteroaggregative E. coli
EHEC: Enterohaemorrhagic E. coli
EIEC: Enteroinvasive E. coli
EPEC: Enteropathogenic E. coli
ETEC: Heat-stable and–labile toxin-producing enterotoxigenic E. coli
Epm: Epimerase
FAP: Familial adenomatous polyposis
Fim: Fimbrial adhesion
HtrA: High temperature requirement A
IBD: Inflammatory bowel diseases
LB: Luria-Bertani
mbeA: DNA mobilization protein A
MOI: Multiplicity-of-infection
MSA: Multiple sequence alignment
oriC: Origin of replication
PCR: Polymerase chain reaction
Phz: Phenazine
pks: Polyketide synthase
UPEC: Uropathogenic E. coli
VFDB: Virulence factor database
