# Supplementary material for: Phenotypic characterization and complete genome of a tumorigenic pathobiont Escherichia coli LI60C3
Source: Gut Pathog. 2025 Aug 20;17:63. doi: 10.1186/s13099-025-00732-1 (PMC12366417; doi:10.1186/s13099-025-00732-1)
Supplement: Supplementary file 1 — Supplementary Material 1 [file 13099_2025_732_MOESM1_ESM.pdf]

Suppl Table 1 Primer pairs of semi-quantitative PCR for bacterial genes

Gene	Oligonucleotide sequence (5'→3')	Size (bp)	Tm (°C)	Ref.
chuA	*F:* GAC GAA CCA ACG GTC AGG AT<br>*R:* TGC CGC CAG TAC CAA AGA CA	279	59	[1, 2]
yjaA	*F:* TGA AGT GTC AGG AGA CGC TG<br>*R:* ATG GAG AAT GCG TTC CTC AAC	211	59	[1, 2]
tspE4.C2	*F:* GAG TAA TGT CGG GGC ATT CA<br>*R:* CGC GCC AAC AAA GTA TTA CG	152	59	[1, 2]
colY	*F:* CCA GTG AAT GTA GGG CTG GT<br>*R:* GGC TGC AAC AAT CTG TCT GA	186	64	This study
mbeA	*F:* GAA CGA CGA GTT CAG GAA GC<br>*R:* GGC AAA CAT CAG CAC TGA GA	226	60	This study
16S rRNA	*F:* CCT ACG GGA GGC AGC AG<br>*R:* ATT ACC GCG GCT GCT GG	194	53	[1, 2]

Footnote: The primer sequences were adapted from the reference of Clermont O et al [1, 2] or designed based on the gene sequence in this study.

Suppl Table 2 Primer pairs of quantitative PCR for bacterial genes

Gene	Oligonucleotide sequence (5'-3')	Size (bp)	Ref.
uidA	*F*: CGG AAG CAA CGC GTA AAC TC	70	[3]
	R: TGA GCG TCG CAG AAC ATT ACA		
capA	*F*: TGT TGG AAA ACG CGG TGA TA	78	This study
	R: CTT CGA AGT GTA AAA GCC ATG TTT AT		
wbgU	*F*: GCGGTTCGATCGCGTAAT	63	This study
	R: GGATTTTCAAGGGAGTAACGTACAC		
dhbF	*F*: CGTGGCCGAGTTGATTTTACA	63	This study
	R: CCGTTGATTAGCGCAGTTTTC		
ehpR	*F*: AGCCCGCAATATACCAATGG	65	This study
	R: GTGCAACAAAAGTGTACCCAAACT		
colY	*F*: TGC GGC AGG CTT TCG A	67	This study
	R: GGC TGC AAC AAT CTG TCT GAC A		
mbeA	*F*: CGC ATT AGA GCA ACT GCA ACA	59	This study
	R: AGG CGC TCT CCC ATT CG		
16S rRNA	*F*: CCT ACG GGA GGC AGC AG	194	[4]
	R: ATT ACC GCG GCT GCT GG		

Footnote: All real-time PCR primers were designed in this study, except those cited from references. The annealing temperature (*Tm*) for all primer pairs is set to 60°C.

References of Supplemental Tables

1. Clermont O, Bonacorsi S, Bingen E. Rapid and simple determination of the Escherichia coli phylogenetic group. Appl Environ Microbiol 2000;66:4555-8.
2. Clermont O, Christenson JK, Denamur E, et al. The Clermont Escherichia coli phylo-typing method revisited: improvement of specificity and detection of new phylo-groups. Environ Microbiol Rep 2013;5:58-65.
3. Walker DI, McQuillan J, Taiwo M, et al. A highly specific Escherichia coli qPCR and its comparison with existing methods for environmental waters. Water Res 2017;126:101-110.
4. Watanabe K, Kodama Y, Harayama S. Design and evaluation of PCR primers to amplify bacterial 16S ribosomal DNA fragments used for community fingerprinting. J Microbiol Methods 2001;44:253-62.
